# Novel Insights Into Gene Signatures and Their Correlation With Immune Infiltration of Peripheral Blood Mononuclear Cells in Behcet’s Disease

**DOI:** 10.3389/fimmu.2021.794800

**Published:** 2021-12-15

**Authors:** Haoting Zhan, Haolong Li, Linlin Cheng, Songxin Yan, Wenjie Zheng, Yongzhe Li

**Affiliations:** ^1^ Department of Clinical Laboratory, Peking Union Medical College Hospital, Peking Union Medical College and Chinese Academy of Medical Sciences, Beijing, China; ^2^ Department, State Key Laboratory of Complex, Severe and Rare Diseases, Peking Union Medical College Hospital, Chinese Academy of Medical Science and Peking Union Medical College, Beijing, China; ^3^ Department of Rheumatology and Clinical Immunology, Peking Union Medical College Hospital, Chinese Academy of Medical Sciences & Peking Union Medical College, National Clinical Research Center for Dermatologic and Immunologic Diseases (NCRC-DID), Ministry of Science & Technology, State Key Laboratory of Complex Severe and Rare Diseases, Key Laboratory of Rheumatology and Clinical Immunology, Ministry of Education, Beijing, China

**Keywords:** Behcet’s disease, gene expression omnibus, biomarkers, diagnosis, immune cell infiltration

## Abstract

**Background:**

Behcet’s disease (BD) is a chronic inflammatory disease that involves systemic vasculitis and mainly manifests as oral and genital ulcers, uveitis, and skin damage as the first clinical symptoms, leading to gastrointestinal, aortic, or even neural deterioration. There is an urgent need for effective gene signatures for BD’s early diagnosis and elucidation of its underlying etiology.

**Methods:**

We identified 82 differentially expressed genes (DEGs) in BD cases compared with healthy controls (HC) after combining two Gene Expression Omnibus datasets. We performed pathway analyses on these DEGs and constructed a gene co-expression network and its correlation with clinical traits. Hub genes were identified using a protein–protein interaction network. We manually selected *CCL4* as a central hub gene, and gene-set enrichment and immune cell subset analyses were applied on patients in high- and low-*CCL4* expression groups. Meanwhile, we validated the diagnostic value of hub genes in differentiating BD patients from HC in peripheral blood mononuclear cells using real-time PCR.

**Results:**

Twelve hub genes were identified, and we validated the upregulation of *CCL4* and the downregulation of *NPY2R* mRNA expression. Higher expression of *CCL4* was accompanied by larger fractions of CD8 + T cells, natural killer cells, M1 macrophages, and activated mast cells. Receiver operator characteristic curves showed good discrimination between cases and controls based on the expression of these genes.

**Conclusion:**

*CCL4* and *NPY2R* could be diagnostic biomarkers for BD that reveal inflammatory status and predict vascular involvement in BD, respectively.

## Introduction

Behcet’s disease (BD), considered a variable vessel vasculitis, is characterized by a wide spectrum of clinical features including mucocutaneous lesions, non-granulomatous uveitis, lower extremity vein/cerebral venous sinus thrombosis, aortic aneurysms, and neurological involvement (1). The diagnostic criteria of the international study group for BD requires oral aphthous as the entry symptom, with the concurrence of genital ulcers, ocular or vascular involvement, as well as pathergy test positivity, which renders an extreme specificity (97.5%) (2). Nonetheless, its dynamic manifestation with disease relapse and remission periods, distinct population and sexual clinical expression, and disease type subsets pose grand challenges in identifying novel biomarkers for the diagnosis of BD.

Genetic risk factors impact BD pathogenesis, as the occurrence of BD is 5.78 times higher in HLA *B51/B5* carriers than in noncarriers (3). BD susceptibility loci, for instance *IL-10*, *STAT*, and *IL-23R* polymorphisms (4, 5), indicate the predominant pathogenetic role of inflammation in the disease etiology. MicroRNA (miRNA), influenced by inflammatory signaling pathways, regulates mRNA expression through pre- and post-translational processes, and miRNA variants have shed light on disease status (6). A recent study in Turkey identified the *FAS* rs1800682 polymorphism and *mir146a* as protective factors against BD (7). Ibrahim and Jadideslam confirmed the association between *mir146a* expression and disease activity, with involvement of the eyes and vasculature (8, 9). Downregulation of *miR-155* and that of *miR146a* likely exert their effects by disturbing Th1/Th17 cell and cytokine homeostasis (10). Profiling analysis of miRNA expression has also identified epigenetic pathways that are enriched in vascular biology, the immune response, and apoptosis (11). Long non-coding RNAs (lncRNAs) are also involved in BD etiology, with the homozygous T allele of rs9517723 in lncRNA *LOC107984558* correlated with ocular and central nervous system (CNS) lesions, the induction of *UBAC2* expression, and overactivation of the ubiquitination-related pathway (12). However, we could not only concentrate on individual gene and ignore the potential interactions between them which might be the backbone of intracellular communications and etiology pathways.

Hub genes are highly connected nodes between co-expression network, exploring of which is vital for seeking diagnostic biomarkers or therapeutic drug targets and clarify the biological regulation process in disease conditions. In this study, we utilized weighted gene co-expression (WGCNA) network and protein-protein interaction (PPI) network to identify hub genes. Since WGCNA analysis could provide a systematic insight into co-expression gene sets and their relationships with clinical phenotypes, we invoked this algorithm to establish a scale-free network using adjacency matrix from which topological overlap matrix and corresponding dissimilarity were converted to differentiate gene co-expression modules (13). For subsequent analysis, we rigorously chose the turquoise module as a candidate module which gains highest correlation coefficient and significant corresponding p value with clinical features among patients suffering Behcet’s disease and healthy controls. Recently, a few of studies have comprehensively utilize WGCNA to filtrate synergistically altered gene sets in autoimmune diseases (14–18). Up till now, amassed structure and function of single protein fragmented our comprehension of their intracellular interactions and pathogenesis pathways. By conducting PPI program, we could easily attain a tighter protein-based co-expressed DEG channel and a more integrated interaction network, the connectivity of which is ranked by combined score calculated from online sources including text-mining, experiments, databases and genomic context information, etc. (19). To predict hub genes bridged through networks, we employed MCODE plugin to visualize intensively related modules and CytoHubba package to screen out hub nodes avail of functional enrichment.

Despite the body of research elucidating the genetic variation contributing to BD, a deep exploration of its molecular mechanisms, interaction networks, and key pathways is lacking. In the present study, we aimed to uncover hub genes acting as biomarkers for the diagnosis, classification, and biological functions and networks of BD using bioinformatics methods and a validation cohort.

## Materials and Methods

### Dataset Acquisition and Normalization

Two microarray profiling datasets from peripheral blood mononuclear cells (PBMCs) (GSE70403 and GSE17114) were downloaded from the Gene Expression Omnibus (GEO) (http://www.ncbi.nlm.nih.gov/geo/) with the screening terms “Behcet Syndrome,” “Triple Symptom Complex,” “Behçet Disease,” “Behcet’s Disease,” “Behcet Disease,” “Adamantiades Behcet Disease,” and the organism “Homo Sapiens.” We merged the two datasets and eliminated batch effects utilizing the R package “sva” (20). Consequently, 14 healthy controls (HCs) from GSE17114 and 56 BD samples (15 from GSE17114 and 41 from GSE70403) were combined.

### Differentially Expressed Gene and Enrichment Analysis

Differentially expressed gene (DEG) analysis was conducted using the “limma” package in R with the thresholds of an adjusted *P* < 0.05 and |log Fold Change (FC)| > 0.5 (21). To identify the biological functions and corresponding pathways involving these DEGs, we performed Gene Ontology (GO) enrichment and visualized the biological process, cellular component (CC), and molecular function (MF) categories and Kyoto Encyclopedia of Genes and Genomes (KEGG) pathway analysis of signaling pathways using the R packages “clusterProfiler” and “enrichplot” (22). The significance cutoff criteria were *P* < 0.05 and FDR < 0.05.

### Identification of Hub Genes and Construction of a Protein–Protein Interaction Network

We clustered PBMC-specific gene modules and established a co-expression network using the R package “WGCNA.” The obvious superiority of WGCNA analysis is to cluster DEGs into co-expression modules and ultimately to identify the specific gene module which is most relevant to clinical phenotype of Behcet’s disease. After shearing samples below the abline (h=20000), we constructed the co-expression network with soft thresholding power to obtain a higher level of scale free R^2^ and mean connectivity (13). In dynamic tree cut and module identification section, we altered 10 as the minimum number of gene modules. Utilizing the clinical traits data containing BD patients and healthy controls from GSE70403 and GSE17114, the gene significance (GS) and module membership (MM) were calculated. The relationship between the gene modules and clinical traits was represented in the form of a heatmap, from which we selected a module that was most clinically relevant.

With a threshold score of 0.150, we constructed a protein–protein interaction (PPI) network consisting of the DEGs using the STRING database (19). We have realized from previous reference (23) that the combined score from STRING database is meant to express an approximate confidence of the association between proteins being true based on every channel of evidence (depended on genomic context information, co-expression, text-mining, experiments and databases). Thus, we have ranked the first 10 interactions by the combined score in the PPI network. To attain a higher degree of connectivity, we applied CytoHubba and then extracted the key Hubba nodes ranked by maximum clique centrality (MCC) in Cytoscape software (24). Using the MCODE plugin (25), we sought dense regions divided by the critical value of a degree cut-off = 2, node score cut-off = 0.2, k-core = 2, and max. Depth = 100.

Furthermore, we united the first 10 genes ranked by the combined score in the PPI network and node genes filtered with an MCC > 5 in CytoHubba analysis, together with genes exported using the MCODE program, and 12 hub genes were ultimately identified for principal component analysis (PCA) in the GSE17114 dataset where Oğuz et al. have divided BD patients into three subtypes containing isolated mucocutaneous manifestations (MB), ocular involvement (OB), and large vein thrombosis (VB) subtypes in accordance with the major clinical symptoms provided by Xavier et al. (26, 27) (detailed methodology is provided in online **Supplementary Materials**).

### Correlation of Hub Genes and Vasculitis

Genecards (https://www.genecards.org/) was screened using the keyword “vasculitis.” We ranked the relevance score given by website and selected top 15 genes, then revisited the normalized gene expression data merged from Method 2.1 to see which genes from Genecards have the intersections with GEO datasets. Thus, we deleted 3 genes which were not documented in normalized gene expression data merged from GEO datasets (*ADA2*, *HLA-DRB*, *C4A*). Finally, the top 12 genes (*PRTN3*, *PTPN22*, *CTLA4*, *DNASE1L3*, *MPO*, *MEFV*, *HLA-B*, *HLA-DPA1*, *HLA-DPB1*, *IL-10*, *TNF* and *CRP*) were selected. According to the relevance score, the top 12 genes were selected. Moreover, we visualized the correlation heatmap using the R package “corrplot” (28).

### Gene-Set Enrichment Analysis

To explore the biological functions of hub genes, we downloaded Gene-Set Enrichment Analysis (GSEA) software (http://software.broadinstitute.org/gsea/index.jsp). The patients were divided into high/low-expression groups according to the median expression of the central hub genes. Afterward, we selected “c2.cp.kegg.v6.1.symbols.gmt” as the reference gene-set in the Molecular Signatures Database (29) and diagrammed the KEGG enrichment plot of distinct expression groups by normalized *P* < 0.05 and FDR < 0.2.

### Correlation Analysis of Gene Signatures and Immune Cells

CYBERSORT is a deconvolution algorithm used to determine the relative proportions of 22 human immune cells from bulk expression data (30). Using the R packages “e1071,” “preprocessCore,” and “limma,” we performed immune cell profiling among the BD samples. Samples with a ***P*** < 0.05 were included. To estimate the diverse immune cell patterns in different central hub gene expression groups, we used “vioplot” in R studio.

### Diagnostic Prediction and Validation of Hub Genes

To validate the expression pattern and diagnostic value of the selected hub genes, we collected blood samples from 16 BD patients and 16 age- and sex-matched HCs under a protocol approved by the Medical Ethics Committee of Peking Union Medical College Hospital with informed consent acquired from the enrolled subjects (demographic and clinical data of the validation population and controls in online **Supplementary Materials**, **Table S3** **)**. We then isolated PBMCs by Ficoll density gradient centrifugation. Further, total RNA was extracted from PBMCs using the Trizol Reagent, and cDNA was reverse-transcribed following the manufacturer’s instructions (Mei5 Biotechnology, Co., Ltd). For amplification, SYBER green (Mei5 Biotechnology, Co., Ltd) was utilized for real-time PCR on a Roche LightCycler 480 system. Finally, the relative expression levels were determined using the 2^−∆∆Ct^ formula after normalization with endogenous *GAPDH* mRNA expression. The primer sequences were as follows: *CCL4* forward CTTTTCTTACACCGCGAGGA and reverse GCTTGCTTCTTTTGGTTTGG, *AGTR2* forward TTCCCTTCCATGTTCTGACC and reverse AAACACACTGCGGAGCTTCT, *NPY2R* forward CAAAACTTCTCCTCCAGTCCCC and reverse AAGGTGGGAGGATCAGAGATGG, *ZIC1* forward GCATCCCAGTTCGCTGCGCAAA and reverse GGAGACACGATGGTGGGAGGCG, and *GADPH* forward CCTCAAGATCATCAGCAAT and reverse CCATCCACAGTCTTCTGGGT. Receiver operator characteristic (ROC) curves were rendered using the R package “pROC,” and DEGs between BD and HC were visualized using “ggpubr.”

## Results

### Screening for DEGs Between BD and HC

**Figure 1:**
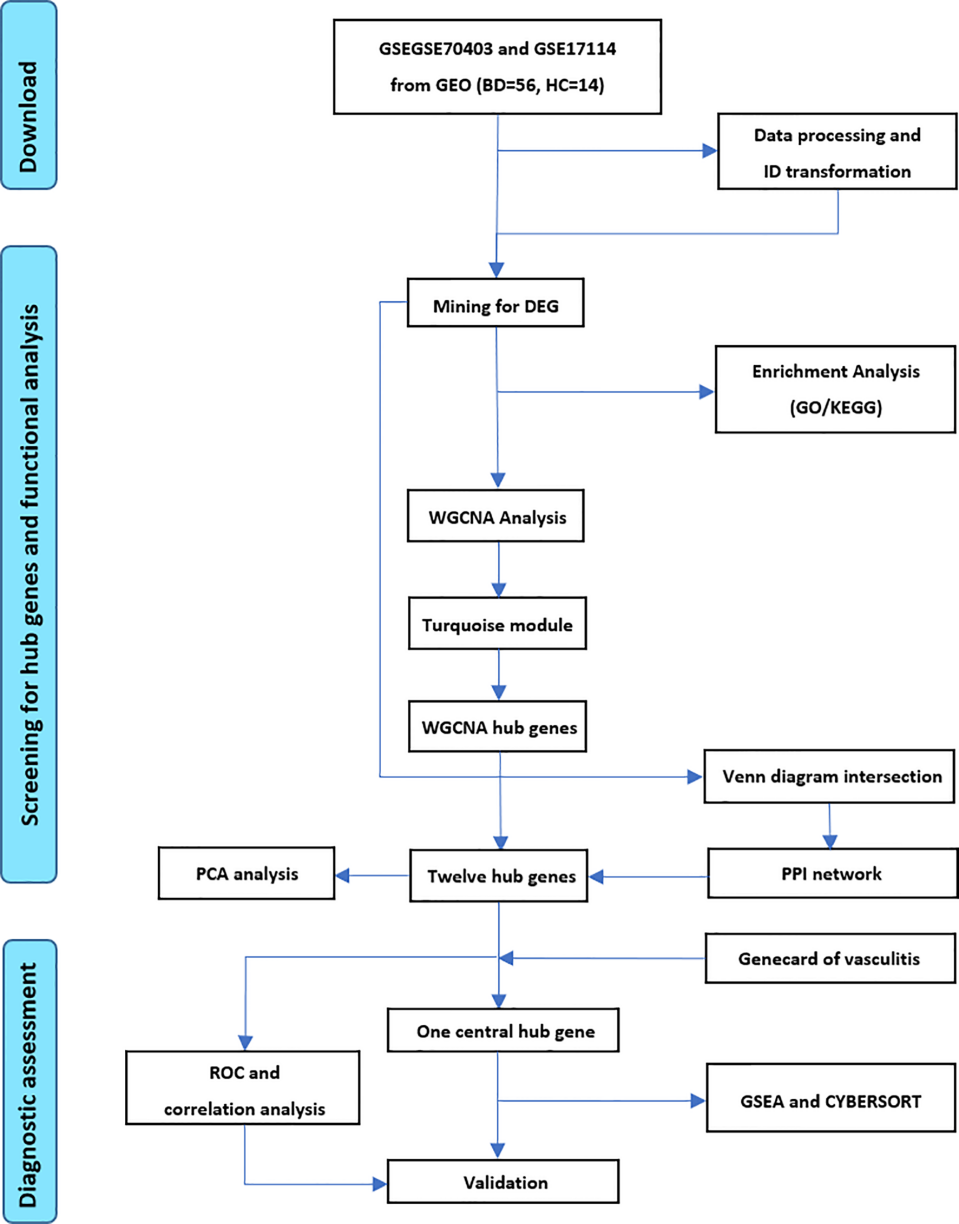
Flow diagram of exploring hub genes and diagnostic markers in BD.

**Figure 2:**
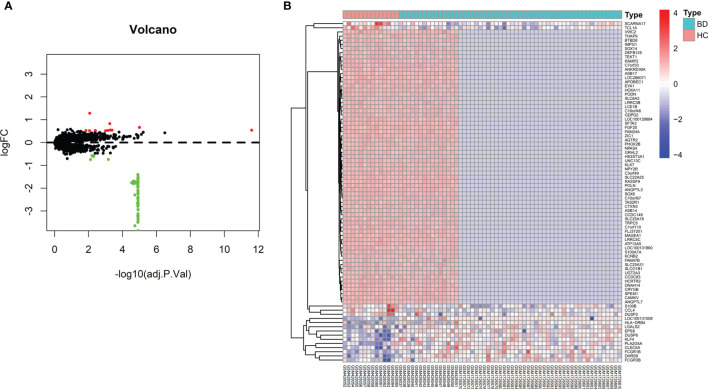
Differentially expressed genes displayed by volcano plot **(A)** and heatmap **(B)**.

The workflow illustrated in **Figure 1** demonstrates the procedure of exploring hub genes and diagnostic markers in BD. Two eligible datasets GSE70403 and GSE17114 with 56 BD and 14 HC combined were included. In total, 82 genes were differentially expressed in BD compared with HC (details provided in **Table S1**). Meanwhile, 11 DEGs were upregulated, and 71 were downregulated (**Figures 2A, B**).

### GO and KEGG Annotation of DEGs

**Figure 3:**
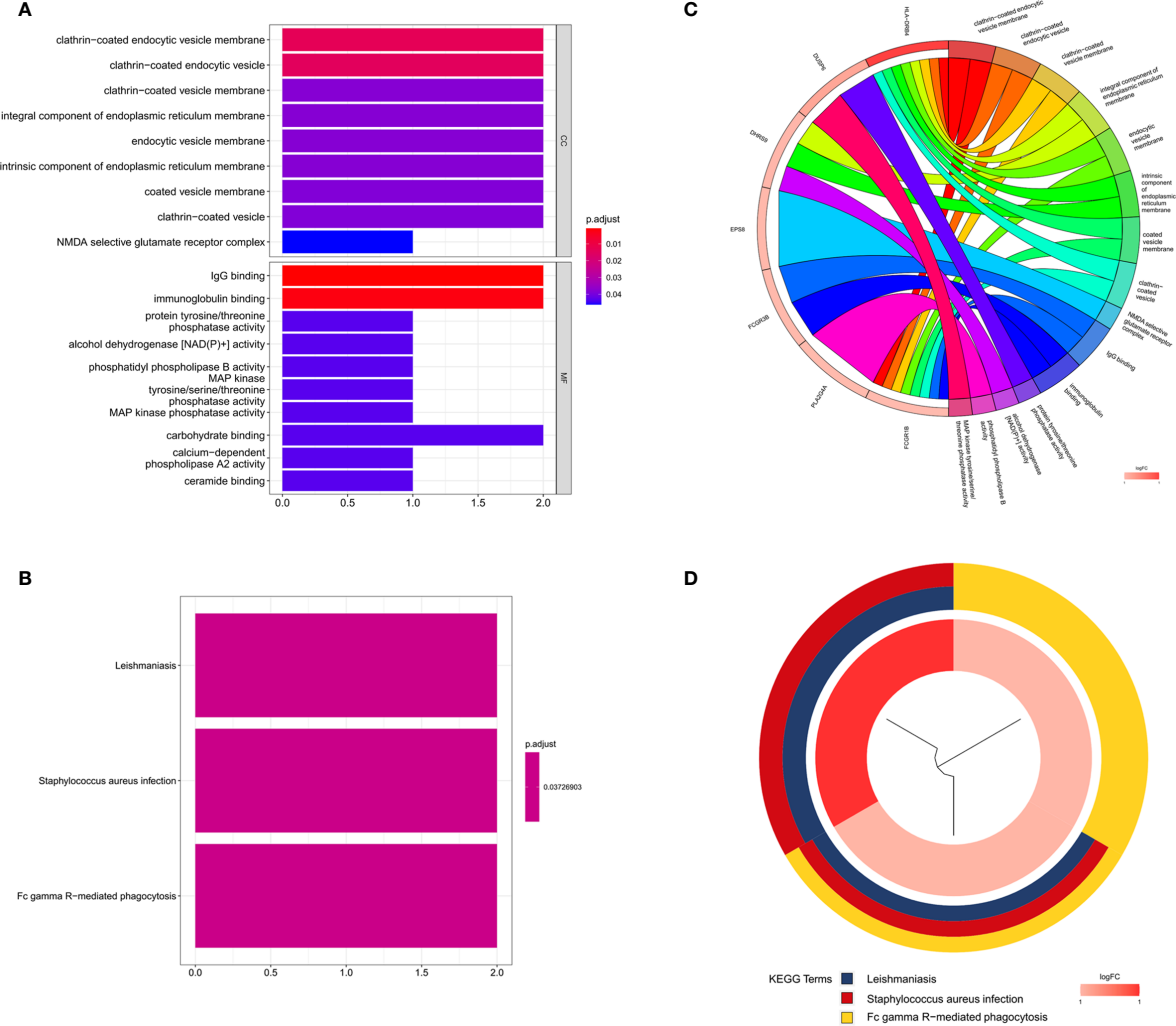
Gene Ontology and KEGG annotation of DEGs **(A)** Histogram of Gene Ontology enrichment analysis: several DEGs were prominently enriched in “clathrin-coated endocytic vesicle membrane” and “clathrin-coated endocytic vesicle” terms in the context of cellular component (CC). For molecular function (MF), DEGs were preferentially included in “IgG binding”, “immunoglobulin binding”, and “carbohydrate binding” which demonstrates the immunopathogenic background of BD. **(B)** Histogram of KEGG pathway analysis: various disease-associated KEGG pathways were hyperactivated, including “Fc gamma R-mediated phagocytosis,” “Staphylococcus aureus infection,” and “Leishmaniasis” which indicates the pathogenesis of BD might be triggered by innate immune activation and immunological response after infection. **(C, D)** Circle plot of Gene Ontology and KEGG annotations.

We investigated the pathogenesis of BD by applying functional and pathway analysis. Several DEGs were prominently enriched in “clathrin-coated endocytic vesicle membrane” and “clathrin-coated endocytic vesicle” terms in the context of CC. For MF, DEGs were preferentially included in “IgG binding,” “immunoglobulin binding,” and “carbohydrate binding” demonstrating the immunopathogenic background of BD (**Figure 3A**). Various disease-associated KEGG pathways were hyperactivated, including “Fc gamma R-mediated phagocytosis,” “Staphylococcus aureus infection,” and “Leishmaniasis” (**Figure 3B**), indicating that the pathogenesis of BD might be triggered by innate immune activation and immunological response after infection (**Figures 3C, D**).

### Identification of Hub Genes

**Figure 4:**
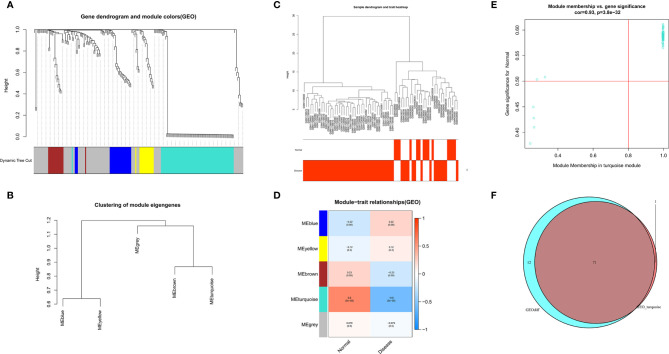
Identification of hub genes **(A)** Gene dendrogram and the modules of DEGs correlated to BD in PBMC of 56 patients and 14 HCs. **(B)** Clustering of module eigengenes **(C)** Sample dendrogram and trait heatmap **(D)** Module-trait relationships: every module has its correlation coefficient and corresponding p value. **(E)** Module membership in turquoise module: the gene significance (GS) and module membership (MM) were calculated and their correlation was 0.93 (*P <*0.001) which indicates the strong relationship between module genes and clinical traits. One dot represents one gene in turquoise module, and dots at upper left corner were highly related to both the turquoise module and clinical traits. **(F)** Venn plot intersecting the DEGs and the turquoise module: there are 71 intersected DEGs for subsequent PPI network analysis.

Five clusters of modules were constructed with WGCNA co-expression network analysis (**Figures 4A, B** and **Table S2**). A sample dendrogram and trait heatmap calculated using Pearson’s correlation coefficients indicated that there were no outlier samples (**Figure 4C**). When incorporating the expression profile with clinical traits, we found that the turquoise module was significantly associated with clinical phenotypes and contained 72 genes (r = 0.60, *P* < 0.05) (**Figures 4D, E**). A Venn plot intersecting the DEGs and the turquoise module narrowed the hub gene candidates down to 71 (**Figure 4F**).

**Figure 5:**
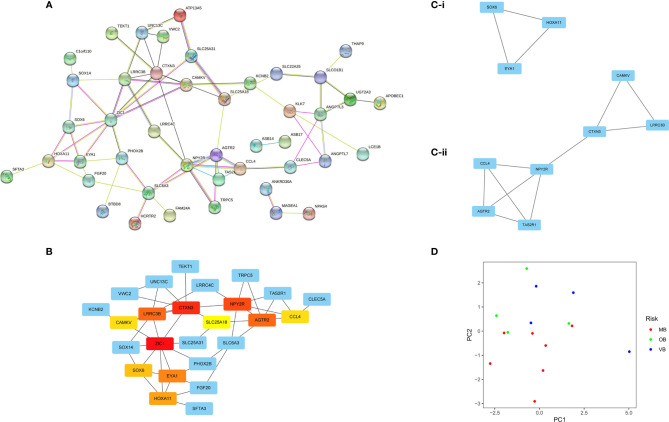
PPI network analysis **(A)** Interrelationship between the proteins encoded by DEGs from STRING database. The thickness of connecting lines suggests the strength of the association between proteins. **(B)** Key Hubba nodes ranked by maximum clique centrality (MCC): the top 10 candidate hub genes ranked by MCC linkage degrees were *ZIC1*, *CTXN3*, *NPY2R*, *AGTR2*, *LRRC3B*, *EYA1*, *HOXA11*, *SOX6*, *CCL4* and *CAMKV*. **(C)** Subnetwork gene nodes exported using the MCODE program: the hub nodes linking highly connected modules were *NPY2R*, *LRRC3B*, *AGTR2*, *CTXN3*, *CAMKV*, *CCL4*, *TAS2R1*, *HOXA11*, *EYA1* and *SOX6*. **(D)** principal component analysis using the GSE17114 dataset: these hub genes could discriminate isolated mucocutaneous manifestations (MB), ocular involvement (OB), and large vein thrombosis (VB) patients diagnosed with BD from in GSE17114.

Next, we input these 71 genes into PPI network analysis to investigate the interrelationship between the proteins encoded by these genes and discovered 70 interactions (**Figure 5A**). We have extracted the first 10 interactions by the combined score in the PPI network. *CCL4, NPY2R, AGTR2, TAS2R1, ASB14, ASB17, C1orf110, SOX14, MAGEA1, NPAS4, EYA1* and *HOXA11* was elected (Set 1). We filtered the top 10 genes ordered by a combined score and an MCC > 5 filtered by CytoHubba, including *ZIC1, CTXN3, NPY2R, AGTR2, LRRC3B, EYA1, HOXA11, SOX6*, *CCL4*, *CAMKV*, *ANGPTL3, TAS2R1, SLC6A3* and *SOX14* (Set 2). (**Figure 5B** and **Table 1**), along with subnetwork gene nodes exported using the MCODE program where *NPY2R, LRRC3B, AGTR2, CTXN3, CAMKV, CCL4* and *TAS2R1* was clustered as subset 1 (shown in **Figure 5C-ii**) and HOXA11, EYA1, SOX6 as subset 2 (shown in **Figure 5C-i**), they are all named as Set 3. (**Figure 5C** and **Table 2**). Eventually, 12 hub genes (*AGTR2, CAMKV, CTXN3, EYA1, HOXA11, LRRC3B, NPY2R, SOX14, SOX6, TAS2R1, ZIC1*, and *CCL4*) were identified (shown in **Figure S1**: a union of the intersection of set 2 and 3 plus the intersection of set 1 and 2). *ZIC1* is the top 1 significant node gene ranked by MCC, therefore we could not neglect it for further analysis and also add it into hub genes. These hub genes were identified for PCA analysis in GSE17114 to divide BD patients based on clinical manifestations (26). These hub genes could well discriminate isolated mucocutaneous manifestations (MB), ocular involvement (OB), and large vein thrombosis (VB) patients diagnosed with BD (**Figure 5D**).

**Table 1:** Hubba nodes ranked by maximum clique centrality (MCC) in CytoHubba.

Node_name	MCC	DMNC	MNC	Degree	EPC	BottleNeck	Ec-Centricity	Closeness	Radiality	Betweenness	Stress	Clusterin Coefficient
***ZIC1***	19	0.26	8	9	21.17	10	0.14773	19.98333	5.971	378.3	712	0.25
***CTXN3***	13	0.32	5	8	21.01	19	0.17727	19.95	6.088	356.5	660	0.18
***NPY2R***	12	0.32	5	7	20.29	16	0.17727	18.51667	5.878	249.9	516	0.24
***LRRC3B***	11	0.32	5	6	20.52	5	0.14773	17.65	5.761	100.2	224	0.33
***AGTR2***	11	0.32	5	6	19.93	1	0.17727	17.18333	5.715	152.5	324	0.33
***EYA1***	10	0.32	5	5	19.31	1	0.12662	15.72619	5.365	25.37	68	0.5
***HOXA11***	9	0.38	4	5	18.75	3	0.12662	15.55952	5.342	89.59	162	0.4
***SOX6***	8	0.38	4	4	18.95	1	0.12662	14.80952	5.248	8	20	0.67
***CAMKV***	7	0.46	3	4	19.08	4	0.17727	16.81667	5.808	190.7	332	0.5
***CCL4***	7	0.46	3	4	18.03	4	0.17727	15.4	5.551	158.7	364	0.5
*ANGPTL3*	6	0.31	3	5	12.97	3	0.14773	14.31667	4.968	131.8	266	0.3
***TAS2R1***	6	0.46	3	3	17.62	1	0.17727	14.16667	5.342	0	0	1
*SLC6A3*	6	0.31	2	6	18.97	4	0.14773	17.1	5.621	249.7	526	0.07
***SOX14***	5	0.31	3	4	18.24	2	0.12662	14.80952	5.248	76.67	178	0.33
*UNC13C*	5	0.31	3	4	18.81	1	0.14773	15.16667	5.411	30	62	0.33
*PHOX2B*	4	0.31	2	4	17.89	5	0.12662	16.02619	5.505	119.5	246	0.17
*ANGPTL7*	4	0.31	3	3	12.36	1	0.14773	12.65	4.782	17.27	58	0.67
*KLK7*	4	0.31	2	4	11.95	5	0.14773	13.56667	5.015	158.5	300	0.17
*SLCO1B1*	4	0.31	2	4	11.75	4	0.14773	13.55	4.945	162.3	298	0.17
*SLC25A18*	4	0	1	4	18.26	8	0.17727	16.86667	5.831	252.9	492	0
*FGF20*	3	0.31	2	3	17.21	1	0.12662	14.10952	5.178	40.1	94	0.33
*SLC25A31*	3	0	1	3	16.73	2	0.17727	15.51667	5.621	87.29	188	0
*CLEC5A*	3	0.31	2	3	13.55	3	0.14773	13.61667	5.132	123.4	292	0.33
*UGT2A3*	3	0.31	2	3	9.913	2	0.12662	11.89524	4.385	74	154	0.33
*SLC22A25*	2	0	1	2	12.23	5	0.17727	13.68333	5.365	175	340	0
*LRRC4C*	2	0	1	2	15.61	1	0.14773	14.43333	5.435	14.9	42	0
*VWC2*	2	0.31	2	2	15.27	1	0.14773	13.58333	5.272	0	0	1
*KCNB2*	2	0	1	2	12.42	3	0.17727	13.65	5.365	152.3	276	0
*ATP13A5*	2	0	1	2	14.06	1	0.14773	12.56667	4.968	2.667	4	0
*MAGEA1*	2	0	1	2	1.989	3	0.06818	2	0.239	2	2	0
*TRPC5*	2	0.31	2	2	14.99	1	0.14773	13.16667	5.155	0	0	1
*THAP9*	1	0	1	1	6.906	1	0.12662	9.79524	4.082	0	0	0
*NPAS4*	1	0	1	1	1.706	1	0.03409	1.5	0.205	0	0	0
*LCE1B*	1	0	1	1	6.932	1	0.12662	9.83571	4.152	0	0	0
*SFTA3*	1	0	1	1	9.689	1	0.1108	10.82738	4.478	0	0	0
*HCRTR2*	1	0	1	1	10.08	1	0.12662	11.56905	4.758	0	0	0
*FAM24A*	1	0	1	1	9.662	1	0.12662	11.56905	4.758	0	0	0
*TEKT1*	1	0	1	1	10.77	1	0.14773	12.91667	5.225	0	0	0
*C1orf110*	1	0	1	1	9.708	1	0.1108	10.51071	4.385	0	0	0
*BTBD8*	1	0	1	1	10	1	0.1108	11.17738	4.642	0	0	0
*ASB17*	1	0	1	1	1.451	1	0.04545	1	0.136	0	0	0
*ASB14*	1	0	1	1	1.451	1	0.04545	1	0.136	0	0	0
*APOBEC1*	1	0	1	1	5.817	1	0.1108	8.88452	3.522	0	0	0
*ANKRD30A*	1	0	1	1	1.785	1	0.03409	1.5	0.205	0	0	0

MCC, Maximal Clique Centrality; DMNC, Density of Maximum Neighborhood Component; MNC, Maximum Neighborhood Component; hey all calculated from local based method. Closeness, EcCentricity, Radiality, Bottle Neck, Stress, Betweenness, Edge percolated Component (EPC) were output in accordance to global-based method. We utilize MCC on behalf of connectivity of hub genes. The bold values represent the hub genes we have selected.

**Table 2:** DEGs ranked by combined score in PPI network.

Node1	Node2	Combined_score
***CCL4***	***NPY2R***	0.922
***AGTR2***	*CCL4*	0.912
*AGTR2*	*NPY2R*	0.905
*AGTR2*	*TAS2R1*	0.9
*ASB14*	*ASB17*	0.9
*CCL4*	***TAS2R1***	0.9
*NPY2R*	*TAS2R1*	0.9
*C1orf110*	***SOX14***	0.536
*MAGEA1*	*NPAS4*	0.503
*EYA1*	*HOXA11*	0.46
*ANKRD30A*	*MAGEA1*	0.361
*EYA1*	*SOX6*	0.356
*SOX6*	*ZIC1*	0.353
*ATP13A5*	*SLC25A31*	0.327
*EYA1*	*FGF20*	0.319
*ATP13A5*	*UNC13C*	0.309
*EYA1*	*ZIC1*	0.305
*CCL4*	*CLEC5A*	0.302
*LRRC3B*	*SOX14*	0.297
*SLC22A25*	*SLC25A18*	0.295
*CAMKV*	*LRRC3B*	0.289
*AGTR2*	*TRPC5*	0.283
*CTXN3*	*LRRC3B*	0.281
*CTXN3*	*ZIC1*	0.276
*SLC25A31*	*ZIC1*	0.27
*ANGPTL3*	*SLCO1B1*	0.258
*SLC22A25*	*SLCO1B1*	0.247
*SLC25A18*	*SLC25A31*	0.235
*KCNB2*	*KLK7*	0.227
*KLK7*	*LCE1B*	0.222
*FGF20*	*SLC6A3*	0.221
*BTBD8*	*PHOX2B*	0.22
*HOXA11*	*SFTA3*	0.219
*PHOX2B*	*ZIC1*	0.218
*SOX14*	*ZIC1*	0.218
*CAMKV*	*CTXN3*	0.213
*NPY2R*	*SLC6A3*	0.213
*FAM24A*	*SLC6A3*	0.211
*AGTR2*	*SLC6A3*	0.208
*LRRC3B*	*LRRC4C*	0.202
*HOXA11*	*SOX6*	0.198
*HCRTR2*	*SLC6A3*	0.197
*CAMKV*	*ZIC1*	0.19
*SLCO1B1*	*UGT2A3*	0.187
*ANGPTL3*	*ANGPTL7*	0.184
*EYA1*	*PHOX2B*	0.181
*CTXN3*	*UNC13C*	0.175
*FGF20*	*HOXA11*	0.173
*HOXA11*	*ZIC1*	0.173
*LRRC3B*	*ZIC1*	0.166
*PHOX2B*	*SLC6A3*	0.166
*ANGPTL3*	*UGT2A3*	0.165
*NPY2R*	*TRPC5*	0.162
*CAMKV*	*KCNB2*	0.161
*SLCO1B1*	*THAP9*	0.16
*SOX14*	*SOX6*	0.16
*CTXN3*	*NPY2R*	0.158
*CTXN3*	*SLC25A18*	0.157
*CTXN3*	*VWC2*	0.157
*ANGPTL3*	*KLK7*	0.156
*ANGPTL3*	*CLEC5A*	0.156
*ANGPTL7*	*KLK7*	0.156
*ANGPTL7*	*CLEC5A*	0.156
*LRRC3B*	*UNC13C*	0.156
*LRRC4C*	*NPY2R*	0.156
*AGTR2*	*SLC25A18*	0.155
*APOBEC1*	*UGT2A3*	0.151
*UNC13C*	*VWC2*	0.151
*CTXN3*	*TEKT1*	0.15

The first 10 interactions by the combined score in the PPI network were CCL4, NPY2R, AGTR2, TAS2R1, ASB14, ASB17, C1orf110, SOX14, MAGEA1, NPAS4, EYA1 and HOXA11 was elected. The bold values represent the hub genes we have selected.

### Correlation Between Hub Genes and Vasculitis

**Figure 6:**
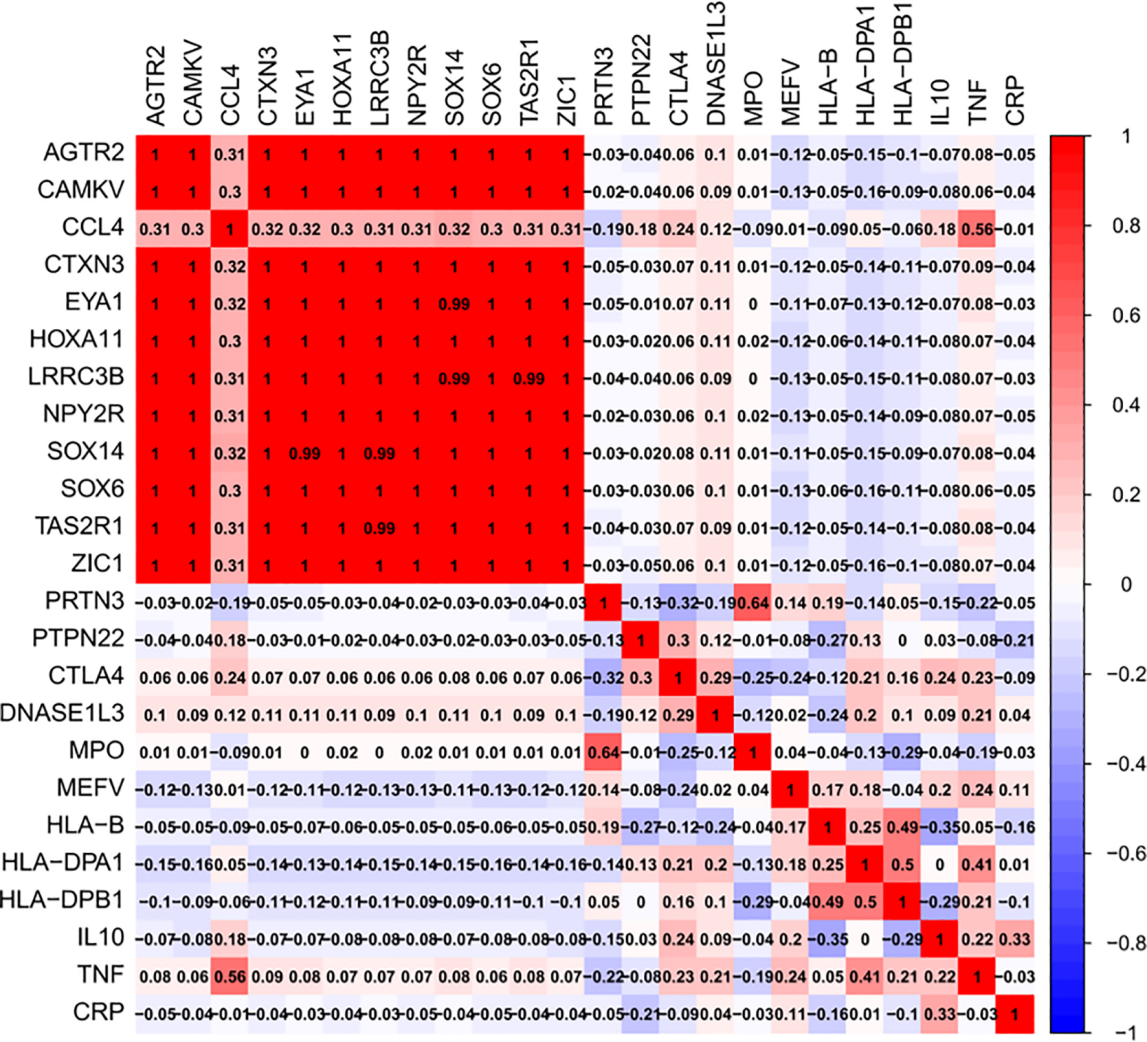
Correlation heatmap between hub Genes and vasculitis. The top 12 genes associated with vasculitis were exported (*PRTN3*, *PTPN22*, *CTLA4*, *DNASE1L3*, *MPO*, *MEFV*, *HLA-B*, *HLA-DPA1*, *HLA-DPB1*, *IL10*, *TNF*, and *CRP*) from Genecards together with 12 hub genes from 12 hub genes (*AGTR2*, *CAMKV*, *CTXN3*, *EYA1*, *HOXA11*, *LRRC3B*, *NPY2R*, *SOX14*, *SOX6*, *TAS2R1*, ZIC1, and *CCL4*) from above WGCNA and PPI analysis.

Genecards was searched, and the top 12 genes associated with vasculitis were exported (*PRTN3*, *PTPN22*, *CTLA4*, *DNASE1L3*, *MPO*, *MEFV*, *HLA-B*, *HLA-DPA1*, *HLA-DPB1*, *IL10*, *TNF*, and *CRP*). The correlation heatmap revealed tight relationships among these 12 hub genes with Pearson’s r ranging from 0.99 to 1 (*P* < 0.05); however, except for *CCL4* and *TNF* (r = 0.56, *P* < 0.05), there was no relationship between the hub genes and the output genes from the Genecards website (**Figure 6**).

### GSEA Analysis of Central Hub Genes

**Figure 7:**
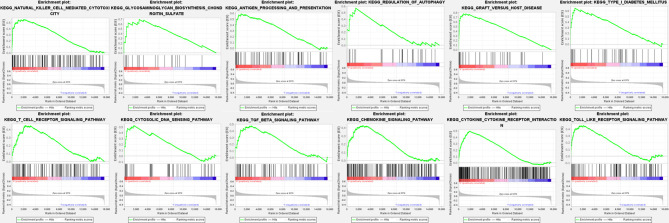
GSEA analysis of central hub genes. BD patients were categorized into high- and low-expression groups of *CCL4* for GSEA function and pathway analysis from which twelve KEGG pathways were enriched.

Through an intensive literature search in PubMed, we selected *CCL4* as the central hub gene because of its known pathogenic role in autoimmune diseases including rheumatoid arthritis (RA) (31), multiple sclerosis (MS) (32) and Behcet’s disease (33). Accordingly, our patients were categorized into high- and low-expression groups of *CCL4* for GSEA function and pathway analysis. Twelve KEGG pathways, “natural killer cell-mediated cytotoxicity,” “glycosaminoglycan biosynthesis chondroitin surface,” “antigen processing and presentation,” “regulation of autophagy,” “graft versus host disease,” “type I diabetes,” “T cell receptor signaling pathway,” “cytosolic DNA sensing pathway,” “TGF-β signaling pathway,” “chemokine signaling pathway,” “cytokine-to-cytokine receptor interaction,” and “toll-like receptor signaling pathway,” were enriched in the *CCL4* high-expression BD patients (**Figure 7**).

### Landscape of Immune Cell Proportions

**Figure 8:**
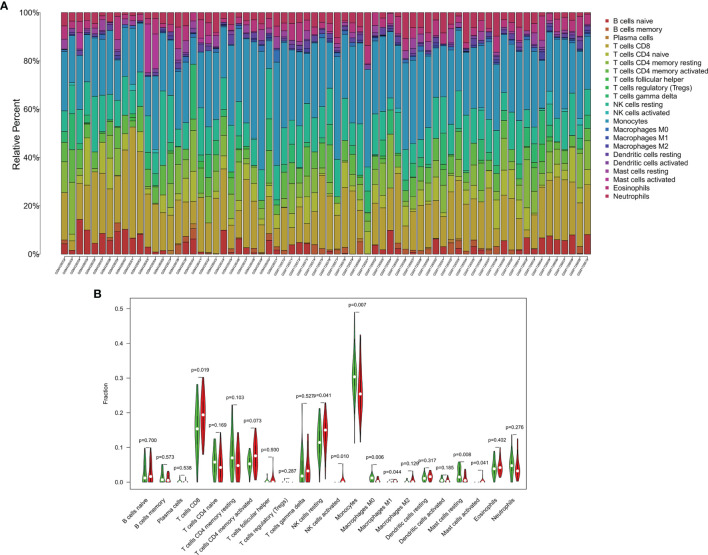
Immune cell proportions among BD patients and HC according to the high/low expression of central hub genes. **(A)** The relative percentage of immune cells are presented. **(B)** Violin plot shows the *CCL4* high-expression group had a higher fraction of CD8 + T cells (*P* = 0.019), Natural killer (NK) cells (both resting and activated, *P* = 0.041 and 0.010, respectively), M1 macrophages (*P* = 0.044), and activated mast cells (*P* = 0.041) in contrast with low-expression group.

We calculated the relative percentage of immune cells among BD patients and HC (**Figure 8A**). In comparison with the low-expression group, the high-expression group had a higher fraction of CD8 + T cells (*P* = 0.019), Natural killer (NK) cells (both resting and activated, *P* = 0.041 and 0.010, respectively), M1 macrophages (*P* = 0.044), and activated mast cells (*P* = 0.041). Inversely, monocytes (*P* = 0.007), M0 macrophages (*P* = 0.006), and resting mast cells (*P* = 0.008) were decreased in BD patients with high *CCL4* expression (**Figure 8B**).

### Diagnostic Performance of Hub Genes and Validation

**Figure 9:**
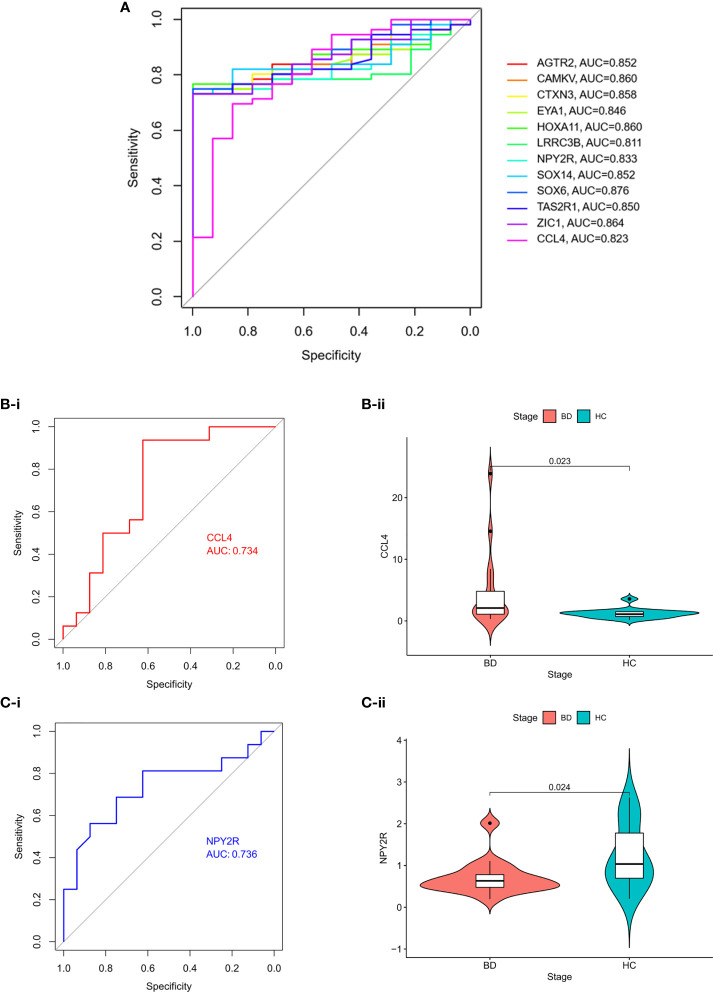
Diagnostic performance of hub genes and validation **(A)** ROC analysis to detect the performance of hub genes in differentiating patients with BD from HC. **(B)** ROC validation and differential expression among BD and HC of *CCL4*. **(C)** ROC validation and differential expression among BD and HC of *NPY2R*.

We then performed ROC analysis to detect the performance of hub genes in differentiating patients with BD from HC. The area under the curve (AUC) ranged from 0.81 to 0.88 (**Figure 9A**). Additionally, *CCL4* was highly expressed in our validation cohort (*P* = 0.0234), whereas *NPY2R* was downregulated (*P* = 0.024) (**Figures 9B, C** and **Table 3**); however, no significant difference was found in *AGTR2* and *ZIC1* expression among BD and HC individuals (data not presented).

**Table 3:** mRNA expression in PBMC of BD and HC from validation cohort.

Type	*CCL4 *(2^-∆∆Ct^)	*NPY2R *(2^-∆∆Ct^)
BD	6.711646	0.962594
BD	1.081725	0.458502
BD	1.236847	2.013911
BD	8.436629	0.719467
BD	1.757267	0.421908
BD	2.439637	0.607097
BD	0.819794	0.481297
BD	0.320116	1.105731
BD	4.167455	0.433769
BD	1.098727	0.501736
BD	14.54549	0.664343
BD	2.360621	0.90125
BD	0.483806	0.659754
BD	2.322749	0.742262
BD	1.873626	0.493116
BD	23.90378	0.195467
HC	0.16647	2.629886
HC	1.41095	1.105731
HC	1.114709	0.895025
HC	1.583738	2.114036
HC	3.563595	0.68302
HC	0.421421	0.779165
HC	0.759611	2.337554
HC	1.498307	0.965936
HC	1.071155	0.203063
HC	1.362101	2.273634
HC	0.492832	0.429283
HC	1.066216	0.461691
HC	1.550249	0.699793
HC	0.539303	1.121166
HC	1.507857	1.664397
HC	1.034667	1.265757

Behcet’s disease, BD; healthy control, HC; peripheral blood mononuclear cell, PBMC.

## Discussion

In this current study, we knit *in-silico* results and the *in vitro* data closely then disclosed the biological functions as well as pathogenic pathways involved in Behçet’s disease (BD). To begin with, we downloaded and merged 2 GEO datasets GSE70403 and GSE17114 by microarray profiling of peripheral blood mononuclear cells from BD patients. WGCNA algorithm identified the most relevant gene set to clinical phenotype of Behçet’s disease which named as turquoise module. Subsequently, PPI network (CytoHubba and MOCDE plugin) was employed to explore the co-expression interactions between DEGs from which hub nodes were extracted (*AGTR2, CAMKV, CTXN3, EYA1, HOXA11, LRRC3B, NPY2R, SOX14, SOX6, TAS2R1, ZIC1*, and *CCL4*). It turns out that these above hub genes could be used to discriminate BD patients into MB, OB, and VB subtypes in accordance with the major clinical symptoms provided by Xavier et al. in GSE 17114 dataset which additionally demonstrated a reliable antidiastole performance (26, 27). Wondering which hub gene is previously considered as the potential pathogenic manipulator for autoimmune disease in references, we retrieved PubMed and only to find *CCL4*, as a central hub gene, stands out in RA, MS and BD etiology. Therefore, we classified the BD patients from microarray datasets following the expression level of *CCL4* for GSEA function, twelve KEGG pathways intensively enriched in intracellular and intercellular biological communications among immune cells. Hence, CYBERSORT was utilized to visualize the immune cell profiling based on the expression level of *CCL4* in BD patients. The upregulated *CCL4* could recruit a higher proportion of CD8 + T cells (*P* = 0.019), NK cells (both resting and activated, *P* = 0.041 and 0.010, respectively), M1 macrophages (*P* = 0.044), and activated mast cells (*P* = 0.041) into inflammatory sites (skin, eyes and even vascular), which likely exacerbates proinflammatory situation, immune cell function and intensify disease activity in BD patients. Last but not least, the performance of hub genes exerts a great ability in discerning BD and HC (AUC ranging from 0.81-0.88) where *CCL4* and *NPY2R* are proved as a moderate diagnostic biomarker in disease recognition from healthy subjects in our validation cohort (clinical characteristics are provided in the online **Supplementary Material**, **Table S1**).

Taken together, we have identified the immune-activated, inflammation-induced, and carbohydrate-mediated pathogenesis of BD and identified DEGs and pathways involved in its etiology. Our results indicate that the identified hub genes could be of diagnostic value and reveal new insights into the etiological analysis of BD.

Subsequent validation disclosed that *CCL4* expression is significantly upregulated in BD patients compared to HC. Additionally, we found that there was a higher proportion of CD8 + T cells, NK cells, M1 macrophages, and activated mast cells infiltrating in inflammation sites in the subset of BD patients with high *CCL4* expression. CCL4 is a proinflammatory chemokine that extensively participates in autoimmune pathogenesis, with immunoregulatory effects on trafficking immune cells into sites of inflammation. For instance, CCL4/MIP-1β secreted by IL-2-activated NK cells amplified IFN-γ release and invoked other inflammatory cells like neutrophils, macrophages, and T cells, which are responsive to chemokines and aggravate the inflammatory process (34). In RA, CCL4 is vital for guiding CD56^dim^CD16^bright^ NK cells into the synovium (35); in addition, it is positively correlated with IL-36 agonists, IL-36RA, and IL-38 cytokines, which intensify joint inflammation (36).

Additionally, CCL4 could indicate the deterioration of BD, as extremely high expression of *CCL4* mRNA was found in patients who suffer from thrombosis and CNS involvement in our validation cohort. A research published by Schecter et al. demonstrated that in human smooth muscle cells enriched in plaque area CCL4/MIP-1β is abundant and introduce the elevation of tissue factor that involves in arterial coagulation together with thrombotic cascade (37). From snap shots of CCL4/MIP-1β participation in clotting formation, there is supposed to exist a hidden mechanism in causing thrombosis of BD patients. Previous evidence supports that upregulated CCL4 could be considered to be a circulating inflammatory cytokine marker in primary and systemic lupus erythematosus (SLE)-related autoimmune hemolytic anemia, indicating erythroid compensation and disease severity due to its positive relationship with reticulocyte count (38). Being a major macrophage attractant, universal involvement of CCL4 has been found in diabetes mellitus, since its circulating concentration increased regardless of disease stage, which may contribute to destruction of islet cells and the progression of diabetes. By providing an inflammatory microenvironment, CCL4 induces cell adhesion to endothelial cells through oxidative stress and tends to augment atherosclerosis and vascular injury (39). Additionally, elevated CCL4/MIP-1β in the cerebrospinal fluid is a characteristic of IgG4 anti‐neurofascin 155-positive chronic inflammatory demyelinating polyneuropathy, which implies exacerbation of protein leakage, damage of encephalic and CCL4/MIP-1β decreases with combined immune-treatment response (40). Della-Torre et al. demonstrated that B cells and fibroblasts might orchestrate inflammatory infiltration through CCL4, which in turn amplified the recruitment of monocytes, CD4 + cytotoxic T cells through CCR5, and IgG4-related disease lesions (41). In discriminating between relapsing–remitting and progressive clinical phenotypes of multiple sclerosis (MS), the plasma CCL4/MIP-1β level showed a good diagnostic value (AUC = 0.702, *P* < 0.0002) and was a protective factor preventing disease progression (odds ratio = 0.9873, *P* = 0.0171) (42). However, the pathogenic impact of CCL4 in BD has not been reported before. As the similar pathogenesis existing among autoimmune diseases, we could deduce that the expression of *CCL4* might be a circulating inflammatory chemokine which aggravates inflammation and disease progression *via* inducing immune cell infiltration and damaging surrounding tissue in BD patients.

Neuropeptide Y receptor subtype 2 (NPY2R) is a G-protein binding receptor for neurotransmitter NPY, which triggers vascular smooth cell proliferation and vasoconstriction, and results in ischemic angiogenesis (43). In our study, *NPY2R* mRNA expression in PBMCs of BD patients, especially in those with severe symptoms including thrombosis and gastrointestinal involvement, was distinctly lower than that in HC. A genome-wide association study demonstrated that the *NPY2R* rs1902491 polymorphism was associated with a severe diabetic retinopathy subgroup without end-stage renal disease (44); however, no significant association was replicated in a small cohort with rapidly proliferative diabetic retinopathy in Lithuania (45). This may imply *NPY2R* is potentially correlated with microvascular leakage and obstruction which could stands for vascular destruction within BD patients. The gene encoding the type 2 angiotensin II (Ang II) receptor (*AGT2R*) plays a pivotal role in atherosclerosis, vascular inflammation, and remodeling and has anti-inflammatory and antiproliferative influences, and it exhibited a female advantage by counteracting the AT1 receptor in mice (46). In SLE, *AGT2R* polymorphisms containing the A allele (CA  +  AA) in exon 3 or G allele (AG  +  GG) in intron 1 are of apparent significance compared with HC (47). *AGT2R* expression is also strongly enhanced in RA synovial tissue, an exogenous agonist that could ameliorate inflammation (48). On the contrary, AGT2R blockers are postulated to dampen the clinical situation and laboratory metrics in RA patients (49). In idiopathic pulmonary fibrosis, *AGT2R* is highly expressed in interstitial fibroblasts and serves as a determinant of fibroblast proliferation and migration (50). In 883 Austrian MS patients and 972 controls. The *ZIC1* rs1841770 polymorphism was associated with an earlier disease onset of MS (51), but a Spanish replication study did not support it as an MS predisposition factor (52). Nevertheless, we did not find any statistical difference in *AGT2R* and *ZIC1* mRNA expression between BD and HC; this may be attributed to the small population of our validation cohort.

NK cells are innate immune lymphocytes that have a cytolytic immune-regulatory effect in autoinflammatory disease, particularly in BD. We confirmed that in patients with upregulated *CCL4* expression, there is a higher fraction of NK cells infiltrating the inflammatory zone. However, conflicting studies have reported decreased, normal, and increased NK cells in BD compared with those in HC (53). It has been hypothesized that impaired NK cytotoxicity and degranulation facilitate and active disease progress (54, 55). The NK1/NK2 ratio is significantly increased, resulting in CD16 + IFN-γ + NK1-induced IFN-γ secretion and suppressed NK regulation in mucocutaneous BD. Moreover, increasing IFN-γ produced by NK cells reflects active disease and relapse of disease, which demonstrated the dominant interaction between NK cells and interferons (56). Accordingly, NKMHC-I modules encoding HLA-B*51 could also contribute to NK cell inhibition through binding killer immunoglobin-like receptors, further supporting hereditary factors as key drivers of BD etiology.

Hyperactivation of monocytes and neutrophils is a feature of BD (57). Our results identified a high level of monocytes in *CCL4* low expression group, instead of neutrophils in BD. Monocytes in BD patients appear to have intensified oxidative burst activity compared with those in RA patients and HC (58). The enriched pathways JAK/STAT, IL-6, and interferon signaling are upregulated in CD14 + monocytes isolated from BD patients (59), whereas toll-like receptors 2, 4, and 5 are highly expressed in monocytes, causing a cascade of activation of nuclear factor-κB and production of TNF-α, unveiling a proinflammatory mechanism of BD (60). Bacterial infection might be a trigger of BD pathogenesis. Lipopolysaccharide (LPS) stimulation increases TNF-α secretion *in vivo* in monocytes of active BD rather than in quiescent ones (61). A recent study demonstrated a perturbed FcγRs balance in monocytes from BD patients that represented downregulated inhibitory FcγRIIb and upregulated activating FcγRIII, the level of which fluctuated along with BD treatment and disease severity. Moreover, LPS and IgG-stimulated monocytes produced more IL-6 than those from HC (57). IgG binding activates FcγRs and FcγRIIb, mainly leading to endocytosis and internalization of immune complexes; therefore, these studies corroborate our finding of “IgG binding,” “clathrin-coated endocytic vesicle membrane,” and “clathrin-coated endocytic vesicle” in our GO analysis and the enriched pathways “Fc gamma R-mediated phagocytosis” and “Staphylococcus aureus infection” in the KEGG analysis.

M1 macrophages stimulate an inflammatory reaction by triggering cytokines such as TNF-α and IL-6, resulting in Th1 reactivity, whereas BD patients with skin lesions conferred M1 macrophage dominance in systemic sclerosis (SSc). It confers that M1 macrophages is more like a predominant pathogenesis factor in BD than in SSc. A higher expression of CCR1 ligands for CCL3/MIP-1α than that in HC again demonstrated that a skewed M1 and M2 macrophage balance led to MIP-1α-induced inflammation (62). Macrophage incubation with active BD serum facilitated CD64 (FcγRI) positivity and *IL-8* mRNA expression compared to inactive BD, suggesting an overwhelming proinflammatory response and aberrant endothelial alteration (63). Mast cells are prominently elevated in the skin lesions of active Behcet’s disease compared with those uninvolved skin (64). It seems that mast cells are attracted by chemotaxis influenced by complement cascades in BD patients with high-expressed *CCL4*, which is analogical to basophil hyperactivation process.

The profound role of IL-17A and CD8 + T cell-mediated immune response is pronounced in BD with papulopustular skin lesions. Investigation into Tc17 cell activation confirmed that CD8 + T cells are infiltrated in peripheral vascular walls and the subcutis in BD, and, concurrently, IL-17A + CD8 + T cells were elevated in BD compared to psoriasis vulgaris lesions and were a major source of IL-17A rather than CD4 + T cells (65). IFN-γ-producing CD8 + T cells are enhanced in the peripheral blood of active BD patients and are even higher in patients with pulmonary manifestations and neurological involvement. Furthermore, the Tc1/Tc2 ratio is dramatically increased in the active and remission phases of BD (66). Increased IFN-γ and CD8 + Leu7 + T cells together with an increased titer of IgG antibodies to HSV-1 demonstrated an antigen-driven T cell-dominant etiology characteristic of BD (67), which is in accordance with our GSEA pathway analysis results.

Our GO analysis highlighted the “carbohydrate binding” pathway, and accumulating evidence suggests that galectins regulate immune cell homeostasis, in which galectin-1 inhibits T cell proliferation, enhances apoptotic activity, and thus suppresses inflammation, whereas galectin-3 amplifies the inflammatory response and survival of T lymphocytes, which subsequentially activates NADPH oxidase, the superoxidative reaction of neutrophils, and recruits monocytes (68). This coincides with our finding of “alcohol dehydrogenase [NAD(P)+] activity” among the MF GO terms. The combination of lectins and epithelial cells and lectins binding to specific tissues triggers antigen humoral responses leading to autoimmune rheumatic responses, for instance, in RA or type I diabetes (69). S-nitrosated mannose binding lectin (SNO-MBL) is upregulated in RA patients and was dysfunctional in depositing C4, phagocytosing bacteria, and binding to apoptotic cells, which consequently caused enhancement of anti-SNO-MBL antibodies (70).

There are several limitations in the present study. First, although we have verified significant difference between BD and HC group in mRNA expressions of *CCL4* and *NPY2R*, we could not associate our results with disease activity index (Behçet’s Disease Current Activity Form) attributed to lack of integrated clinical information of each patient. Second, we aimed to identify a diagnostic marker in discerning BD patients and healthy subjects, thus we did not add disease controls such as takayasu arteritis, inflammatory bowel disease, ANCA-associated systemic vasculitis and even patients with either coronary or venous thrombosis which shares semblable clinical feature with BD. Third, *via* MCODE program we have discovered the protein interaction network between *CCL4* and *NPY2R*, future analysis should more concentrate on underlying up/down regulation between them which might be the mechanism leading to vasculitis moreover vascular deteriorations in BD. Further studies using more comprehensive data and larger validation cohort should be performed to elucidate the association of *CCL4* and *NPY2R* with BD activity as well as clinical phenotypes.

In conclusion, as a proinflammatory factor synthesized in the inflammation zone, higher mRNA expression of *CCL4* might be an upstream switch to attract more immune cells into inflammatory sites, these cells in turn positively amplify cytokine secretion and exacerbates BD disease stage. In vasculitis related to BD, *NPY2R* expression is potentially impaired and may be a marker of vascular complications. Future studies are required to elucidate the potential mechanisms underlying the dysregulated expression of target genes and their correlation with the immune microenvironment and clinical phenotypes in BD.

## Data Availability Statement

The original contributions presented in the study are included in the article/**Supplementary Material**. Further inquiries can be directed to the corresponding author.

## Ethics Statement

The studies including human participants were approved by Medical Ethics Committee of Peking Union Medical College Hospital with informed consensus acquired from enrolled subjects. The patients/participants provided their written informed consent to participate in this study.

## Author Contributions

YL conceived and designed the research. HZ extracted data, performed software analysis and visualized graphs and tables. LC, SY, HL, and HZ categorized and supervised graphs and tables. WZ provided the clinical data of each patient. HZ and HL went on validation experiments. HZ wrote the paper. All authors are accountable for all aspects of the study, and attest to the accuracy and integrity of the results. All authors contributed to the article and approved the submitted version.

## Funding

This research was supported by grants from the National Key Research and Development Program of China (2018YFE0207300), the National Natural Science Foundation of China Grants (81871302,81871299) and Beijing Key Clinical Specialty for Laboratory Medicine - Excellent Project (No. ZK201000).

## Conflict of Interest

The authors declare that the research was conducted in the absence of any commercial or financial relationships that could be construed as a potential conflict of interest.

## Publisher’s Note

All claims expressed in this article are solely those of the authors and do not necessarily represent those of their affiliated organizations, or those of the publisher, the editors and the reviewers. Any product that may be evaluated in this article, or claim that may be made by its manufacturer, is not guaranteed or endorsed by the publisher.
