# Supplementary material for: Novel Insights Into Gene Signatures and Their Correlation With Immune Infiltration of Peripheral Blood Mononuclear Cells in Behcet’s Disease
Source: Front Immunol. 2021 Dec 15;12:794800. doi: 10.3389/fimmu.2021.794800 (PMC8714896; doi:10.3389/fimmu.2021.794800)
Supplement: Supplementary file 1 [file DataSheet_1.pdf]

Supplementary Materials

Supplementary Methodology

The procedures of selecting hub genes were following the flow chart presented in Figure 1 rigorously.

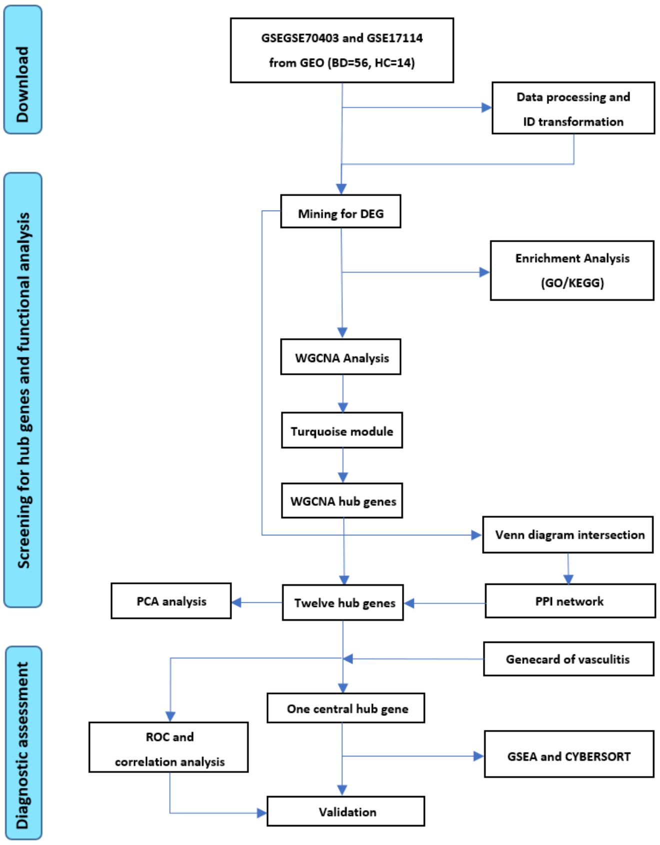

Figure 1 Flow diagram of exploring hub genes and diagnostic markers in BD

First and foremost, we screened GEO datasets from http://www.ncbi.nlm.nih.gov/geo/ using search terms **(("Behcet Syndrome"[Mesh]) OR (Triple Symptom Complex [Title/Abstract] OR Behçet Disease[Title/Abstract] OR Adamantiades Behcet Disease[Title/Abstract] OR Behcet's Disease[Title/Abstract] OR Behcet Disease[Title/Abstract])) AND "Homo sapiens"[porgn:__txid9606]**. Only to find 2 datasets met the requirement of microarray profiling of peripheral blood mononuclear cells from Behçet's disease (BD) patients, we downloaded **GSE70403 and GSE17114** for subsequent data processing and ID transformation.

Secondly, we **merged** these two datasets and **eliminated batch effect**s utilizing the R package "sva". As a result, 14 healthy controls (HCs) from GSE17114 and 56 BD samples (15 from GSE17114 and 41 from GSE70403) were combined.

Thirdly, we mined for differentially expressed genes (DEGs) using R/limma package with the threshold value of **adjusted P < 0.05 and |log Fold Change (FC)| > 0.5**. The reason why we chose the cut off value of **|log$_2$ Fold Change (FC)| > 0.5** is that we aimed to filtered more DEGs using a

relatively lower fold change value. We made our prudent choice after reading literatures previously published and found that most of them preferred to set **log2 fold change ≥ 0.585 (fold change ≥ 1.5) with a false discovery rate (FDR) < 0.05** (1-3) or **log2 Fold Change (FC) ≥ 0.5 (fold change ≥ 1.4) with *p* value < 0.05 (4)** to enlarge the screening criteria.

Fourthly, we aimed to identify modules and genes for Behcet's disease using weighted gene co-expression network analysis (WGCNA). The obvious superiority of WGCNA analysis is to cluster DEGs into co-expression modules and ultimately to identify the specific gene module which is most relevant to clinical phenotype of Behcet's disease. After shearing samples below the abline (h=20000), we constructed the co-expression network with soft thresholding power to obtain a higher level of scale free R^2 and mean connectivity. In dynamic tree cut and module identification section, we altered 10 as the minimum number of gene modules. Utilizing the clinical traits data containing BD patients and healthy controls from GSE70403 and GSE17114, the gene significance (GS) and module membership (MM) were calculated and presented in **Figure 4E,** the correlation of GS and MM was 0.93 (***P*** <0.001) which indicates the strong relationship between module genes and clinical traits. In our study, we chose **turquoise module** on account of **its highest correlation coefficient and significant corresponding p value (shown in Figure 4D)**.

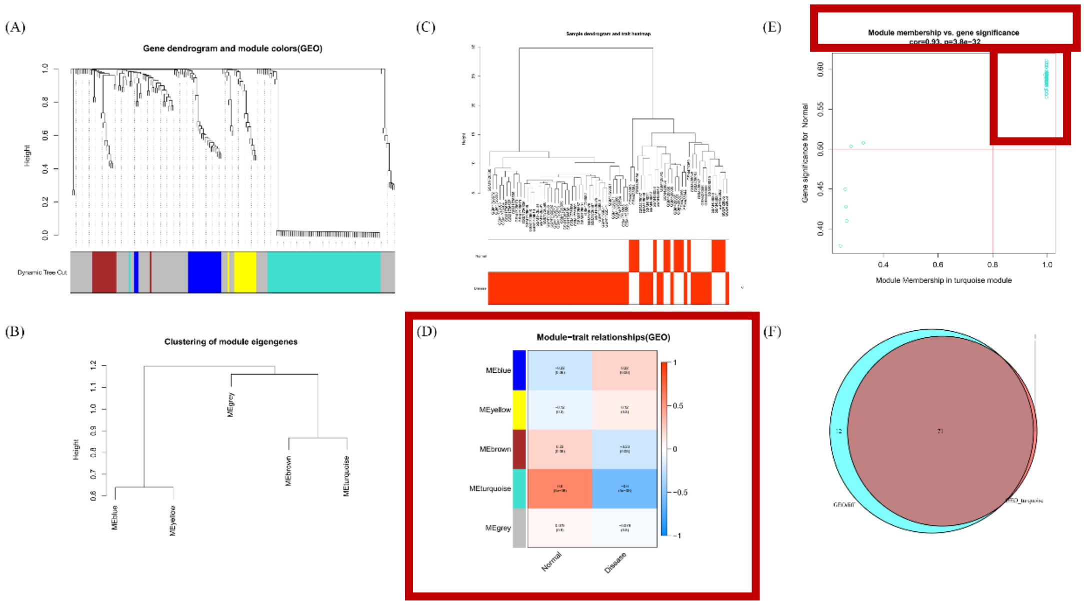

Fifthly, we intersected DEGs and the turquoise module narrowed the hub gene candidates down to 71 for further analysis.

(1) We sacrificed high statistical confidence for a more expansive protein–protein interaction (PPI) network using the **threshold score of 0.150** in STRING database.

(2) We have realized from previous reference (5) that the ***combined score from STRING database is meant to express an approximate confidence of the association between proteins being true*** based on every channel of evidence (seven independent 'channels': three prediction channels based

on genomic context information, and one channel each for i. co-expression, ii. text-mining, iii. biochemical/genetic data called 'experiments' and iv. previously curated pathway and protein-complex knowledge called 'databases'). Thus, we have **ranked the first 10 interactions** by the combined score in the PPI network. *CCL4, NPY2R, AGTR2, TAS2R1, ASB14, ASB17, C1orf110, SOX14, MAGEA1, NPAS4, EYA1* and *HOXA11* was elected **(Set 1)**.

(3) The Maximal Clique Centrality (MCC) algorithm was invoked for the sake of hub genes with CytoHubba plugin, in our study, the **top 10** candidate hub genes ranked by MCC linkage degrees were identified(6-8). Then we extracted the node genes filtered with **MCC > 5**, including *ZIC1, CTXN3, NPY2R, AGTR2, LRRC3B, EYA1, HOXA11, SOX6*, *CCL4*, *CAMKV*, *ANGPTL3, TAS2R1, SLC6A3* and *SOX14* **(Set 2)**.

(4) Molecular Complex Detection (MCODE) plugin was optimized to find significant modules with **a degree cut-off = 2, node score cut-off = 0.2, k-core = 2, and max. Depth = 100** as threshold value (9, 10), eventually, *NPY2R, LRRC3B, AGTR2, CTXN3, CAMKV, CCL4* and *TAS2R1* was clustered as subset 1 (shown in **Figure 5C-ii**) and HOXA11, EYA1, SOX6 as subset 2 (shown in **Figure 5C-i**), they are all named as **Set 3**.

(5) Finally, we united the **first 10 interactions ranked by the combined score in the PPI network** (Set 1: *CCL4, NPY2R, AGTR2, TAS2R1, ASB14, ASB17, C1orf110, SOX14, MAGEA1, NPAS4, EYA1* and *HOXA11*) and **significant node genes filtered with MCC > 5 in CytoHubba** analysis (Set 2: *ZIC1, CTXN3, NPY2R, AGTR2, LRRC3B, EYA1, HOXA11, SOX6*, *CCL4*, *CAMKV*, *ANGPTL3, TAS2R1, SLC6A3* and *SOX14*), together with **subset genes exported using the MCODE program** (Set 3: *NPY2R, LRRC3B, AGTR2, CTXN3, CAMKV, CCL4, TAS2R1, HOXA11, EYA1, SOX6*), and **12 hub genes (*AGTR2, CAMKV, CTXN3, EYA1, HOXA11, LRRC3B, NPY2R, SOX14, SOX6, TAS2R1, ZIC1,* and *CCL4*)** were identified (shown in figure below: **a union of** the **intersection of set 2 and 3 plus the intersection of set 1 and 2**). ***ZIC1*** is the top 1 significant node gene ranked by MCC, therefore we could not neglect it for further analysis and also add it into hub genes.

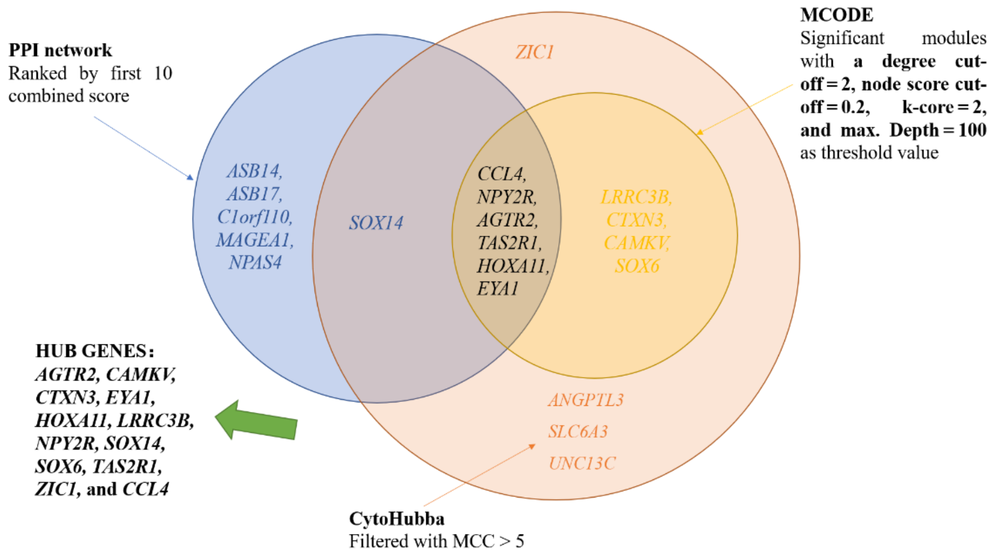

Figure S1: identification of hub genes

Table S1 Differentially expressed genes in BD compared with HC

Gene	Regulation	logFC	AveExpr	t	***P***.Value	adj.***P***.Val	B
LOC100131508	Up	0.541559	0.909806	10.68013	1.56E-16	2.45E-12	26.6817
DUSP6	Up	0.665589	3.895009	6.744978	3.17E-09	9.92E-06	10.86339
SLCO1B1	Down	-2.11786	1.507261	-6.57237	6.56E-09	1.19E-05	10.17503
SLC25A18	Down	-2.44091	1.69064	-6.53308	7.74E-09	1.19E-05	10.01885
TEKT1	Down	-3.2859	2.163816	-6.50027	8.89E-09	1.19E-05	9.888606
S100A7A	Down	-2.7327	1.861875	-6.47646	9.82E-09	1.19E-05	9.794201
LOC100129884	Down	-2.92926	1.971124	-6.46697	1.02E-08	1.19E-05	9.756558
LOC286071	Down	-3.11376	2.077515	-6.44239	1.13E-08	1.19E-05	9.65922
SLC25A31	Down	-1.46828	1.162355	-6.42098	1.24E-08	1.19E-05	9.574472
TRPC5	Down	-1.57233	1.228337	-6.35237	1.65E-08	1.19E-05	9.303414
CCDC83	Down	-1.56221	1.222591	-6.32563	1.85E-08	1.19E-05	9.197981
SOX6	Down	-2.68876	1.863055	-6.31467	1.93E-08	1.19E-05	9.154779
HOXA11	Down	-2.94355	2.006796	-6.31104	1.96E-08	1.19E-05	9.140481
LRRC4C	Down	-2.22564	1.599644	-6.30789	1.99E-08	1.19E-05	9.12807
ATP13A5	Down	-2.44525	1.726216	-6.29646	2.08E-08	1.19E-05	9.08305
KCNB2	Down	-2.56597	1.796359	-6.29001	2.14E-08	1.19E-05	9.057684
DNAH14	Down	-1.63654	1.269495	-6.28889	2.15E-08	1.19E-05	9.053262
HCRTR2	Down	-2.46409	1.735858	-6.2838	2.20E-08	1.19E-05	9.033255
PHOX2B	Down	-2.75404	1.903115	-6.28136	2.22E-08	1.19E-05	9.02366
ZIC1	Down	-2.21144	1.596795	-6.27484	2.28E-08	1.19E-05	8.998011
C10orf67	Down	-3.35576	2.247332	-6.27459	2.28E-08	1.19E-05	8.997006
VWC2	Down	-1.52565	1.201959	-6.26511	2.37E-08	1.19E-05	8.959735
CAMKV	Down	-3.22919	2.177464	-6.25371	2.49E-08	1.19E-05	8.914916
APOBEC1	Down	-2.24802	1.619096	-6.2473	2.56E-08	1.19E-05	8.88974
SFTA3	Down	-1.53432	1.215874	-6.24497	2.58E-08	1.19E-05	8.880596
PODN	Down	-3.4398	2.300918	-6.24331	2.60E-08	1.19E-05	8.874082
C1orf110	Down	-1.57839	1.241003	-6.23005	2.75E-08	1.19E-05	8.821981
IMPG1	Down	-1.55035	1.225811	-6.2299	2.75E-08	1.19E-05	8.821398
RAMP2	Down	-3.2843	2.213251	-6.22601	2.79E-08	1.19E-05	8.806143
ANGPTL3	Down	-1.71228	1.321158	-6.21893	2.88E-08	1.19E-05	8.778338
POLN	Down	-3.20342	2.172727	-6.21128	2.97E-08	1.19E-05	8.748364
MAGEA1	Down	-2.37532	1.696944	-6.21069	2.98E-08	1.19E-05	8.746022
ANGPTL7	Down	-2.64098	1.850794	-6.20501	3.05E-08	1.19E-05	8.723762
SLC6A3	Down	-3.86786	2.553135	-6.20205	3.09E-08	1.19E-05	8.712134
TAS2R1	Down	-3.19499	2.168204	-6.20018	3.11E-08	1.19E-05	8.704804
THAP9	Down	-2.71679	1.89275	-6.18781	3.27E-08	1.19E-05	8.656319
AGTR2	Down	-1.90176	1.431571	-6.18432	3.32E-08	1.19E-05	8.642669
LOC100131860	Down	-1.92037	1.442064	-6.18157	3.36E-08	1.19E-05	8.631886
KLK7	Down	-3.25041	2.204966	-6.18134	3.36E-08	1.19E-05	8.631003
FGF20	Down	-1.76974	1.356729	-6.17652	3.43E-08	1.19E-05	8.612129
UNC13C	Down	-1.79651	1.377275	-6.13641	4.05E-08	1.23E-05	8.455162
SPEM1	Down	-3.49904	2.354139	-6.13205	4.12E-08	1.23E-05	8.438119

NPY2R	Down	-2.09488	1.5496	-6.13109	4.14E-08	1.23E-05	8.434365
DEFB125	Down	-1.91877	1.445263	-6.12544	4.24E-08	1.23E-05	8.412319
EYA1	Down	-2.79861	1.953656	-6.12535	4.24E-08	1.23E-05	8.411958
NPAS4	Down	-2.72419	1.911496	-6.12417	4.26E-08	1.23E-05	8.407346
C7orf33	Down	-2.13237	1.571502	-6.12294	4.28E-08	1.23E-05	8.402522
UGT2A3	Down	-1.4009	1.149968	-6.12256	4.29E-08	1.23E-05	8.401049
SOX14	Down	-1.97415	1.479675	-6.11774	4.38E-08	1.23E-05	8.38222
CTXN3	Down	-1.85891	1.414099	-6.11478	4.43E-08	1.23E-05	8.370662
ASB14	Down	-2.42706	1.743093	-6.11127	4.49E-08	1.23E-05	8.356944
CRYGB	Down	-3.20645	2.192751	-6.10458	4.62E-08	1.25E-05	8.330824
C3orf49	Down	-2.22741	1.629021	-6.10012	4.71E-08	1.25E-05	8.313415
C16orf46	Down	-2.62463	1.861029	-6.08437	5.02E-08	1.25E-05	8.251986
RASSF9	Down	-1.58829	1.26292	-6.08371	5.04E-08	1.25E-05	8.249417
GRHL2	Down	-1.87007	1.4249	-6.07861	5.14E-08	1.25E-05	8.229535
FAM24A	Down	-2.45768	1.765922	-6.07595	5.20E-08	1.25E-05	8.219152
HS3ST3A1	Down	-1.6961	1.326632	-6.06607	5.42E-08	1.28E-05	8.180681
BTBD8	Down	-1.69701	1.325625	-6.06182	5.51E-08	1.29E-05	8.164092
SLC22A25	Down	-2.67831	1.897937	-6.05685	5.63E-08	1.29E-05	8.144745
ANKRD30A	Down	-1.7331	1.353363	-6.00497	6.97E-08	1.56E-05	7.942998
FAM47B	Down	-1.78411	1.383164	-5.98182	7.66E-08	1.69E-05	7.853099
GDPD2	Down	-3.64826	2.481955	-5.95529	8.54E-08	1.85E-05	7.75025
LCE1B	Down	-2.29646	1.689431	-5.95304	8.62E-08	1.85E-05	7.741558
LRRC3B	Down	-1.70573	1.343748	-5.94013	9.09E-08	1.92E-05	7.691537
CCDC148	Down	-1.80894	1.411395	-5.89381	1.10E-07	2.26E-05	7.512475
ASB17	Down	-1.69277	1.342399	-5.88605	1.13E-07	2.31E-05	7.482508
FLJ37201	Down	-1.7623	1.390751	-5.82844	1.44E-07	2.88E-05	7.260505
DHRS9	Up	0.543273	2.634629	5.09996	2.62E-06	0.000406	4.525065
KLF4	Up	0.548011	3.822384	5.024314	3.51E-06	0.000528	4.24986
LGALS2	Up	0.822981	4.016321	5.00705	3.75E-06	0.000559	4.187316
CCL4	Down	-0.73812	4.128475	-4.94582	4.75E-06	0.000682	3.966304
FCGR3B	Up	0.527706	3.566038	4.928579	5.07E-06	0.000722	3.904314
FCGR1B	Up	0.518552	3.829046	4.826245	7.50E-06	0.000994	3.538488
EPS8	Up	0.53206	2.353709	4.355254	4.30E-05	0.003932	1.906597
SCARNA17	Down	-0.57729	3.606171	-4.26993	5.84E-05	0.004913	1.620932
DUSP2	Down	-0.61523	3.746005	-4.23697	6.57E-05	0.005326	1.511438
S100B	Down	-0.57477	1.41613	-4.17269	8.25E-05	0.006243	1.299393
TCL1A	Down	-0.74281	3.087034	-4.07324	0.000117	0.0076	0.97514
HLA-DRB4	Up	1.282484	2.631385	4.027297	0.000137	0.008472	0.826971
PLA2G4A	Up	0.519551	2.170147	4.012915	0.000144	0.008723	0.780804
CLEC5A	Up	0.523327	2.442555	3.812111	0.000287	0.014099	0.147171

Table S2 Module membership (MM) and gene significance (GS) for WGCNA analysis

Gene	Module Color	GS.HC	***P*.GS.HC**	GS.BD	***P*.GS.BD**	MMblue	***P*.MMblue**	MMyellow	***P*.MM yellow**	MM brown	***P*.MM brown**	MM turquoise	***P*.MM turquoise**	MMgrey	***P*.MMgrey**
ALAS2	blue	-0.1392	0.2506	0.1392	0.2506	0.8901	0.0000	0.2983	0.0121	-0.2148	0.0741	-0.0887	0.4653	-0.2093	0.0820
CA1	blue	-0.2821	0.0180	0.2821	0.0180	0.8958	0.0000	0.3662	0.0018	-0.2661	0.0260	-0.1795	0.1370	-0.1995	0.0977
CD69	blue	0.0634	0.6018	-0.0634	0.6018	-0.5788	0.0000	-0.2695	0.0241	0.1266	0.2963	0.0172	0.8879	0.5476	0.0000
CENPK	blue	-0.0107	0.9302	0.0107	0.9302	-0.5322	0.0000	-0.3342	0.0047	0.2370	0.0482	-0.0353	0.7716	0.0932	0.4429
EPB42	blue	-0.2156	0.0731	0.2156	0.0731	0.9570	0.0000	0.3539	0.0027	-0.1790	0.1381	-0.1341	0.2684	-0.2497	0.0371
GMPR	blue	-0.1338	0.2695	0.1338	0.2695	0.9466	0.0000	0.2861	0.0164	-0.2191	0.0684	-0.0842	0.4882	-0.2261	0.0598
HBD	blue	-0.2630	0.0278	0.2630	0.0278	0.9393	0.0000	0.3924	0.0008	-0.2542	0.0337	-0.1681	0.1643	-0.1903	0.1146
HBM	blue	-0.2972	0.0125	0.2972	0.0125	0.8913	0.0000	0.2610	0.0291	-0.2298	0.0556	-0.1808	0.1342	-0.1957	0.1045
HBQ1	blue	-0.3319	0.0050	0.3319	0.0050	0.8340	0.0000	0.3079	0.0095	-0.1548	0.2008	-0.1984	0.0996	-0.1946	0.1065
HBZ	blue	-0.3539	0.0027	0.3539	0.0027	0.5196	0.0000	0.0628	0.6057	-0.0650	0.5927	-0.2198	0.0675	-0.2486	0.0380
HEMGN	blue	-0.1440	0.2344	0.1440	0.2344	0.8593	0.0000	0.3725	0.0015	-0.2736	0.0219	-0.1028	0.3969	-0.2557	0.0326
KRT1	blue	-0.2307	0.0547	0.2307	0.0547	0.8236	0.0000	0.1305	0.2815	-0.2182	0.0695	-0.1451	0.2308	-0.2071	0.0854
PDZK1IP1	blue	-0.1736	0.1508	0.1736	0.1508	0.6483	0.0000	0.1465	0.2261	-0.0729	0.5487	-0.1035	0.3939	-0.1589	0.1888
PF4	blue	0.0139	0.9089	-0.0139	0.9089	0.4548	0.0001	0.2773	0.0201	-0.1323	0.2748	-0.0146	0.9046	-0.0886	0.4658
PPIL4	blue	-0.1045	0.3894	0.1045	0.3894	-0.4840	0.0000	-0.1931	0.1093	0.0869	0.4742	-0.0871	0.4731	0.3778	0.0013
RGS1	blue	0.1083	0.3721	-0.1083	0.3721	-0.5643	0.0000	-0.3391	0.0041	0.0986	0.4165	0.0569	0.6402	0.6559	0.0000
RNF182	blue	-0.0051	0.9663	0.0051	0.9663	0.4195	0.0003	0.2536	0.0341	-0.1855	0.1243	-0.0830	0.4943	-0.0288	0.8131
SELENBP1	blue	-0.1467	0.2256	0.1467	0.2256	0.9325	0.0000	0.2874	0.0158	-0.1286	0.2888	-0.0969	0.4248	-0.2444	0.0415
SLC25A39	blue	-0.2182	0.0696	0.2182	0.0696	0.9130	0.0000	0.2502	0.0367	-0.1217	0.3155	-0.1424	0.2397	-0.3237	0.0063
SLC4A1	blue	-0.1639	0.1750	0.1639	0.1750	0.9496	0.0000	0.3269	0.0057	-0.2534	0.0343	-0.1063	0.3811	-0.2087	0.0829
SNCA	blue	-0.2201	0.0671	0.2201	0.0671	0.9454	0.0000	0.3482	0.0031	-0.2239	0.0624	-0.1337	0.2699	-0.2558	0.0326
STRADB	blue	-0.2535	0.0342	0.2535	0.0342	0.9560	0.0000	0.3773	0.0013	-0.2402	0.0452	-0.1629	0.1779	-0.2689	0.0244
TRIM58	blue	-0.2426	0.0430	0.2426	0.0430	0.9184	0.0000	0.3540	0.0026	-0.2829	0.0176	-0.1511	0.2118	-0.1959	0.1041

XK	blue	-0.1339	0.2693	0.1339	0.2693	0.8377	0.0000	0.3062	0.0099	-0.1475	0.2229	-0.0873	0.4723	-0.2647	0.0268
BANK1	brown	0.2048	0.0891	-0.2048	0.0891	-0.3060	0.0100	-0.2318	0.0535	0.9338	0.0000	0.1178	0.3315	-0.2535	0.0342
DACT1	brown	0.3161	0.0077	-0.3161	0.0077	-0.1138	0.3481	-0.3283	0.0055	0.5211	0.0000	0.1792	0.1378	-0.1009	0.4060
DSP	brown	0.0152	0.9003	-0.0152	0.9003	-0.1383	0.2534	-0.1257	0.2997	0.5120	0.0000	-0.0796	0.5124	-0.0273	0.8222
FCRL1	brown	0.3062	0.0099	-0.3062	0.0099	-0.3441	0.0035	-0.1991	0.0985	0.8921	0.0000	0.1771	0.1425	-0.1824	0.1308
FCRLA	brown	0.2160	0.0725	-0.2160	0.0725	-0.2749	0.0213	-0.2253	0.0608	0.9336	0.0000	0.1282	0.2902	-0.2260	0.0599
IGHD	brown	0.3217	0.0066	-0.3217	0.0066	-0.2099	0.0812	-0.1611	0.1827	0.8886	0.0000	0.1874	0.1203	-0.2732	0.0221
IGHM	brown	0.2108	0.0798	-0.2108	0.0798	-0.1742	0.1492	-0.2097	0.0815	0.8598	0.0000	0.1183	0.3294	-0.3386	0.0041
IGJ	brown	0.0085	0.9443	-0.0085	0.9443	-0.3635	0.0020	-0.1494	0.2171	0.3941	0.0007	-0.0188	0.8773	0.0600	0.6218
IL7	brown	-0.1113	0.3592	0.1113	0.3592	-0.3387	0.0041	-0.1766	0.1435	0.4543	0.0001	-0.0663	0.5856	0.1171	0.3342
KCNJ2	brown	0.0245	0.8404	-0.0245	0.8404	-0.0179	0.8833	0.1299	0.2838	-0.6243	0.0000	0.0050	0.9672	0.4947	0.0000
MS4A1	brown	0.2950	0.0132	-0.2950	0.0132	-0.2456	0.0404	-0.2644	0.0270	0.9525	0.0000	0.1677	0.1652	-0.3584	0.0023
PFKFB3	brown	0.2391	0.0462	-0.2391	0.0462	-0.1995	0.0978	-0.0273	0.8223	-0.5669	0.0000	0.1312	0.2791	0.6684	0.0000
TCL1A	brown	0.4323	0.0002	-0.4323	0.0002	-0.2783	0.0196	-0.2978	0.0123	0.9135	0.0000	0.2560	0.0324	-0.1772	0.1423
TNFAIP6	brown	0.0359	0.7682	-0.0359	0.7682	0.0117	0.9235	0.2411	0.0444	-0.6563	0.0000	-0.0006	0.9958	0.4551	0.0001
TNFRSF17	brown	0.1098	0.3657	-0.1098	0.3657	-0.2421	0.0435	-0.0747	0.5390	0.5216	0.0000	0.0570	0.6394	-0.2593	0.0302
VPREB3	brown	0.2875	0.0158	-0.2875	0.0158	0.0452	0.7099	-0.2367	0.0485	0.8508	0.0000	0.1752	0.1468	-0.3534	0.0027
ACCS	grey	0.1024	0.3990	-0.1024	0.3990	-0.1355	0.2633	-0.1470	0.2247	0.0464	0.7027	0.0595	0.6244	0.1739	0.1499
APOBEC3B	grey	0.0071	0.9538	-0.0071	0.9538	-0.2956	0.0130	-0.0964	0.4271	0.0991	0.4143	-0.0074	0.9516	-0.1334	0.2709
AREG	grey	0.1780	0.1405	-0.1780	0.1405	-0.3160	0.0077	0.0881	0.4685	-0.4095	0.0004	0.0682	0.5750	0.5178	0.0000
BTNL3	grey	0.0356	0.7701	-0.0356	0.7701	-0.0861	0.4783	-0.0566	0.6419	0.2174	0.0706	0.0269	0.8253	-0.0394	0.7459
C15orf48	grey	-0.1558	0.1979	0.1558	0.1979	-0.1544	0.2020	-0.0346	0.7764	-0.3219	0.0066	-0.1062	0.3817	0.6266	0.0000
C17orf97	grey	-0.2364	0.0488	0.2364	0.0488	0.1844	0.1264	0.2184	0.0693	-0.2959	0.0129	-0.1413	0.2433	0.0302	0.8043
C4BPA	grey	-0.0803	0.5090	0.0803	0.5090	0.0135	0.9119	0.1351	0.2648	-0.2944	0.0134	-0.0558	0.6467	0.1947	0.1063
CCL20	grey	0.2379	0.0474	-0.2379	0.0474	-0.2275	0.0582	-0.1497	0.2160	-0.3346	0.0046	0.1300	0.2835	0.7965	0.0000
CD79A	grey	0.3371	0.0043	-0.3371	0.0043	0.4005	0.0006	0.1067	0.3792	0.4010	0.0006	0.1910	0.1133	-0.3237	0.0063

CD83	grey	0.0714	0.5570	-0.0714	0.5570	-0.3440	0.0035	-0.3774	0.0013	0.1496	0.2165	0.0326	0.7885	0.7360	0.0000
CDKN1C	grey	-0.3637	0.0020	0.3637	0.0020	0.1090	0.3692	-0.2461	0.0400	0.1894	0.1162	-0.2172	0.0709	-0.1208	0.3190
CLC	grey	-0.0671	0.5810	0.0671	0.5810	0.0387	0.7503	0.2827	0.0177	0.2067	0.0861	-0.0629	0.6047	-0.0946	0.4358
CLEC12B	grey	-0.2388	0.0465	0.2388	0.0465	0.0458	0.7066	0.2500	0.0369	-0.3892	0.0009	-0.1711	0.1568	0.0333	0.7844
CPA3	grey	0.0830	0.4945	-0.0830	0.4945	-0.0121	0.9205	0.2730	0.0222	0.1206	0.3199	0.0422	0.7286	-0.0358	0.7683
CXCL1	grey	0.0882	0.4677	-0.0882	0.4677	-0.3045	0.0104	-0.0855	0.4817	-0.3775	0.0013	0.0448	0.7126	0.8141	0.0000
CXCL2	grey	-0.0144	0.9055	0.0144	0.9055	-0.2062	0.0868	-0.0663	0.5853	-0.3715	0.0015	-0.0178	0.8840	0.8754	0.0000
CXCL3	grey	0.0953	0.4325	-0.0953	0.4325	-0.0679	0.5763	-0.1142	0.3466	-0.4098	0.0004	0.0432	0.7227	0.8190	0.0000
DDX3Y	grey	0.0163	0.8932	-0.0163	0.8932	-0.1871	0.1208	0.2289	0.0567	-0.0571	0.6385	-0.0310	0.7990	-0.0301	0.8044
DUSP2	grey	0.4467	0.0001	-0.4467	0.0001	-0.2521	0.0353	-0.2545	0.0335	0.0177	0.8843	0.2499	0.0370	0.5166	0.0000
EGR1	grey	-0.1326	0.2738	0.1326	0.2738	0.1251	0.3021	-0.0739	0.5430	-0.0554	0.6489	-0.0923	0.4470	0.4089	0.0004
EGR2	grey	-0.2155	0.0732	0.2155	0.0732	-0.1775	0.1416	-0.1878	0.1194	-0.0212	0.8619	-0.1358	0.2624	0.6181	0.0000
EIF1AY	grey	0.0186	0.8786	-0.0186	0.8786	-0.1973	0.1016	0.2446	0.0413	-0.0606	0.6185	-0.0031	0.9800	-0.0457	0.7070
ERAP2	grey	-0.1625	0.1790	0.1625	0.1790	-0.1262	0.2980	0.0112	0.9266	0.2213	0.0657	-0.1064	0.3808	0.0986	0.4167
EREG	grey	0.2578	0.0312	-0.2578	0.0312	-0.3204	0.0069	-0.1007	0.4066	-0.2973	0.0125	0.1368	0.2586	0.7624	0.0000
FAM3B	grey	-0.1512	0.2116	0.1512	0.2116	0.2535	0.0342	0.3071	0.0097	-0.2954	0.0131	-0.1841	0.1270	0.0433	0.7217
FFAR2	grey	-0.1008	0.4063	0.1008	0.4063	0.1551	0.1999	0.0127	0.9168	-0.3948	0.0007	-0.0608	0.6171	0.5177	0.0000
FOLR3	grey	-0.0061	0.9602	0.0061	0.9602	0.1955	0.1049	0.3641	0.0019	-0.3231	0.0064	-0.0169	0.8895	-0.0062	0.9596
FOSB	grey	-0.1005	0.4076	0.1005	0.4076	-0.1315	0.2779	-0.1765	0.1439	-0.0947	0.4353	-0.0758	0.5326	0.6009	0.0000
G0S2	grey	0.2206	0.0665	-0.2206	0.0665	-0.2911	0.0145	-0.1644	0.1737	-0.1271	0.2946	0.1232	0.3095	0.7531	0.0000
GPR183	grey	0.0740	0.5428	-0.0740	0.5428	-0.4682	0.0000	-0.3910	0.0008	0.1092	0.3680	0.0195	0.8729	0.6778	0.0000
GPR34	grey	-0.1686	0.1630	0.1686	0.1630	-0.1198	0.3234	0.2338	0.0514	0.0055	0.9642	-0.1091	0.3685	-0.0837	0.4911
HDC	grey	0.0628	0.6055	-0.0628	0.6055	-0.0863	0.4776	0.2994	0.0118	0.0709	0.5597	0.0228	0.8514	0.0105	0.9313
HIST1H1E	grey	0.2582	0.0309	-0.2582	0.0309	-0.1378	0.2553	-0.1333	0.2712	-0.0033	0.9783	0.1520	0.2092	0.0456	0.7079
HLA-DQA1	grey	-0.0816	0.5020	0.0816	0.5020	0.0299	0.8058	-0.1049	0.3873	0.1201	0.3220	-0.0756	0.5339	0.1276	0.2925
HLA-DQB1	grey	-0.1194	0.3249	0.1194	0.3249	0.1048	0.3877	-0.1941	0.1073	0.0804	0.5083	-0.0808	0.5061	0.0729	0.5489

ID1	grey	-0.1072	0.3771	0.1072	0.3771	-0.1206	0.3199	0.0027	0.9821	-0.1809	0.1340	-0.0655	0.5899	0.5332	0.0000
IER3	grey	0.0546	0.6536	-0.0546	0.6536	0.0472	0.6981	0.0609	0.6163	-0.3227	0.0064	0.0141	0.9081	0.7253	0.0000
IFI44L	grey	-0.1877	0.1198	0.1877	0.1198	-0.0759	0.5323	-0.0706	0.5612	-0.2203	0.0668	-0.1267	0.2960	0.2417	0.0438
IFNG	grey	-0.1284	0.2895	0.1284	0.2895	-0.3529	0.0027	-0.2157	0.0730	0.0535	0.6602	-0.0829	0.4950	0.4814	0.0000
IL18RAP	grey	0.2296	0.0559	-0.2296	0.0559	-0.2072	0.0853	0.1119	0.3565	-0.3628	0.0020	0.1287	0.2882	0.1534	0.2049
IL1B	grey	0.0541	0.6564	-0.0541	0.6564	-0.2311	0.0542	-0.1688	0.1625	-0.3327	0.0049	0.0229	0.8506	0.9184	0.0000
JUN	grey	-0.1739	0.1500	0.1739	0.1500	0.0199	0.8701	0.0140	0.9085	0.0333	0.7844	-0.1175	0.3327	0.4664	0.0000
KLRC3	grey	0.2906	0.0147	-0.2906	0.0147	-0.3661	0.0018	-0.2379	0.0473	0.0376	0.7573	0.1630	0.1777	0.1330	0.2723
KLRC4	grey	0.0996	0.4119	-0.0996	0.4119	-0.3380	0.0042	-0.1312	0.2789	0.1354	0.2637	0.0541	0.6563	0.1010	0.4055
LIPC	grey	-0.2351	0.0501	0.2351	0.0501	-0.1112	0.3595	-0.0349	0.7745	-0.0661	0.5865	-0.1470	0.2245	0.0806	0.5069
LRRN3	grey	0.2436	0.0422	-0.2436	0.0422	-0.2334	0.0518	-0.1871	0.1208	0.4271	0.0002	0.1280	0.2911	-0.1861	0.1230
LYZ	grey	-0.2790	0.0193	0.2790	0.0193	-0.2536	0.0342	-0.0455	0.7081	-0.0775	0.5237	-0.2002	0.0965	0.0689	0.5707
MAFF	grey	0.1748	0.1477	-0.1748	0.1477	-0.0511	0.6746	-0.1910	0.1133	-0.3070	0.0097	0.0922	0.4479	0.6204	0.0000
MS4A2	grey	-0.1001	0.4096	0.1001	0.4096	-0.0771	0.5259	0.2587	0.0306	0.1339	0.2691	-0.0924	0.4470	0.0529	0.6638
MYOM2	grey	0.2624	0.0282	-0.2624	0.0282	0.0235	0.8470	-0.1150	0.3431	-0.0488	0.6884	0.1549	0.2003	0.1053	0.3855
NR4A2	grey	0.2225	0.0641	-0.2225	0.0641	-0.3241	0.0062	-0.0509	0.6757	-0.2235	0.0629	0.1184	0.3291	0.7716	0.0000
OSM	grey	0.0854	0.4823	-0.0854	0.4823	-0.2542	0.0337	-0.0793	0.5138	-0.3955	0.0007	0.0485	0.6899	0.7710	0.0000
PF4V1	grey	-0.1070	0.3779	0.1070	0.3779	0.1129	0.3522	0.3235	0.0063	-0.1434	0.2362	-0.0777	0.5225	0.0881	0.4683
PLK2	grey	-0.0835	0.4921	0.0835	0.4921	-0.3904	0.0008	-0.0319	0.7930	-0.3520	0.0028	-0.0646	0.5953	0.7846	0.0000
PPP1R15A	grey	0.0504	0.6787	-0.0504	0.6787	-0.0210	0.8628	-0.1091	0.3686	-0.1638	0.1753	0.0065	0.9576	0.7188	0.0000
PRKY	grey	0.0238	0.8452	-0.0238	0.8452	-0.3190	0.0071	0.0621	0.6094	0.0897	0.4603	0.0049	0.9678	-0.0440	0.7178
PSPH	grey	0.0445	0.7144	-0.0445	0.7144	0.0335	0.7833	-0.0487	0.6888	0.1001	0.4096	-0.0102	0.9331	-0.0024	0.9841
PTGS2	grey	0.1387	0.2522	-0.1387	0.2522	-0.3731	0.0015	-0.1257	0.2998	-0.3036	0.0106	0.0669	0.5820	0.9164	0.0000
PTX3	grey	0.1506	0.2133	-0.1506	0.2133	-0.3917	0.0008	-0.0256	0.8336	-0.3306	0.0052	0.0804	0.5080	0.8224	0.0000
RPS4Y1	grey	0.0447	0.7135	-0.0447	0.7135	-0.1459	0.2281	0.1951	0.1055	-0.0204	0.8671	0.0200	0.8697	-0.0765	0.5290
S100B	grey	0.4414	0.0001	-0.4414	0.0001	-0.3372	0.0043	-0.1963	0.1033	0.1052	0.3861	0.2041	0.0902	0.3066	0.0098

SERPINB2	grey	-0.1174	0.3332	0.1174	0.3332	-0.1451	0.2308	0.0911	0.4531	-0.4314	0.0002	-0.1301	0.2830	0.6609	0.0000
SGK1	grey	-0.2502	0.0367	0.2502	0.0367	-0.2148	0.0741	-0.0344	0.7776	-0.2618	0.0286	-0.1762	0.1445	0.7290	0.0000
SNX7	grey	-0.1927	0.1099	0.1927	0.1099	0.2897	0.0150	0.1650	0.1723	-0.2292	0.0564	-0.1176	0.3321	0.0792	0.5148
THBS1	grey	0.2609	0.0291	-0.2609	0.0291	0.0221	0.8559	-0.0221	0.8560	-0.4717	0.0000	0.1488	0.2190	0.5338	0.0000
TMEM176A	grey	0.0743	0.5408	-0.0743	0.5408	0.1226	0.3118	-0.0779	0.5216	-0.0264	0.8280	0.0270	0.8244	-0.0555	0.6484
TMEM176B	grey	0.0405	0.7393	-0.0405	0.7393	0.0426	0.7262	-0.1071	0.3777	0.0341	0.7794	0.0164	0.8930	-0.0440	0.7173
TMTC1	grey	-0.1342	0.2680	0.1342	0.2680	0.0567	0.6410	0.2345	0.0507	-0.1918	0.1116	-0.0888	0.4647	0.0496	0.6833
TNF	grey	0.1046	0.3889	-0.1046	0.3889	-0.1016	0.4028	-0.2495	0.0373	-0.1068	0.3788	0.0607	0.6176	0.7090	0.0000
TNFAIP3	grey	0.3271	0.0057	-0.3271	0.0057	-0.3375	0.0043	-0.2533	0.0343	-0.0295	0.8088	0.1747	0.1481	0.7041	0.0000
TTTY15	grey	0.2305	0.0549	-0.2305	0.0549	-0.2308	0.0546	0.1787	0.1389	-0.0423	0.7282	0.1315	0.2779	0.0302	0.8040
TUBB2A	grey	-0.3145	0.0080	0.3145	0.0080	0.2294	0.0560	0.2315	0.0539	-0.1237	0.3078	-0.1999	0.0970	0.1958	0.1043
USP9Y	grey	0.0603	0.6200	-0.0603	0.6200	-0.2084	0.0834	0.1951	0.1056	-0.0583	0.6314	-0.0228	0.8515	-0.0640	0.5986
UTS2	grey	-0.1093	0.3679	0.1093	0.3679	-0.0928	0.4448	0.0313	0.7970	0.0245	0.8406	-0.1095	0.3667	-0.0204	0.8670
VNN1	grey	0.0681	0.5754	-0.0681	0.5754	-0.1632	0.1770	0.2930	0.0138	-0.3655	0.0019	0.0265	0.8274	0.1776	0.1413
XIST	grey	-0.1204	0.3209	0.1204	0.3209	0.1375	0.2563	-0.2429	0.0428	0.0792	0.5147	-0.1278	0.2917	0.0581	0.6328
ZFY	grey	-0.0611	0.6156	0.0611	0.6156	-0.1623	0.1795	0.3264	0.0058	-0.1939	0.1077	-0.1661	0.1693	-0.0156	0.8978
AGTR2	turquoise	0.5880	0.0000	-0.5880	0.0000	-0.1254	0.3011	-0.0987	0.4164	0.1198	0.3234	0.9983	0.0000	0.0283	0.8161
ANGPTL3	turquoise	0.5902	0.0000	-0.5902	0.0000	-0.1387	0.2521	-0.1063	0.3813	0.1352	0.2646	0.9991	0.0000	0.0254	0.8347
ANGPTL7	turquoise	0.5891	0.0000	-0.5891	0.0000	-0.1199	0.3227	-0.1029	0.3966	0.1320	0.2762	0.9983	0.0000	0.0294	0.8089
ANKRD30A	turquoise	0.5767	0.0000	-0.5767	0.0000	-0.1444	0.2329	-0.0912	0.4526	0.1068	0.3787	0.9959	0.0000	0.0626	0.6066
APOBEC1	turquoise	0.5918	0.0000	-0.5918	0.0000	-0.1238	0.3073	-0.1003	0.4088	0.1341	0.2682	0.9969	0.0000	0.0082	0.9464
ASB14	turquoise	0.5833	0.0000	-0.5833	0.0000	-0.1340	0.2687	-0.1170	0.3347	0.1276	0.2925	0.9988	0.0000	0.0160	0.8957
ASB17	turquoise	0.5690	0.0000	-0.5690	0.0000	-0.1363	0.2607	-0.0912	0.4527	0.1308	0.2803	0.9964	0.0000	0.0240	0.8434
ATP13A5	turquoise	0.5948	0.0000	-0.5948	0.0000	-0.1491	0.2178	-0.1040	0.3914	0.1289	0.2874	0.9985	0.0000	0.0213	0.8612
BTBD8	turquoise	0.5803	0.0000	-0.5803	0.0000	-0.1239	0.3067	-0.0935	0.4412	0.1388	0.2518	0.9954	0.0000	-0.0061	0.9603
C10orf67	turquoise	0.5934	0.0000	-0.5934	0.0000	-0.1430	0.2376	-0.1044	0.3898	0.1389	0.2516	0.9996	0.0000	0.0181	0.8820

C16orf46	turquoise	0.5816	0.0000	-0.5816	0.0000	-0.1308	0.2803	-0.1098	0.3656	0.1291	0.2870	0.9978	0.0000	0.0305	0.8020
C1QA	turquoise	-0.3785	0.0012	0.3785	0.0012	0.1403	0.2466	-0.0148	0.9033	-0.2275	0.0582	-0.2426	0.0431	0.0471	0.6985
C1orf110	turquoise	0.5909	0.0000	-0.5909	0.0000	-0.1489	0.2185	-0.1119	0.3565	0.1187	0.3278	0.9970	0.0000	0.0270	0.8241
C3orf49	turquoise	0.5826	0.0000	-0.5826	0.0000	-0.1345	0.2668	-0.1067	0.3791	0.1256	0.3003	0.9984	0.0000	0.0242	0.8422
C7orf33	turquoise	0.5841	0.0000	-0.5841	0.0000	-0.1494	0.2170	-0.1034	0.3942	0.1275	0.2930	0.9976	0.0000	0.0504	0.6785
CAMKV	turquoise	0.5921	0.0000	-0.5921	0.0000	-0.1301	0.2832	-0.1034	0.3943	0.1306	0.2810	0.9984	0.0000	0.0173	0.8873
CCDC148	turquoise	0.5695	0.0000	-0.5695	0.0000	-0.1352	0.2643	-0.1057	0.3840	0.1269	0.2952	0.9977	0.0000	0.0287	0.8132
CCDC83	turquoise	0.5968	0.0000	-0.5968	0.0000	-0.1325	0.2742	-0.1057	0.3840	0.1332	0.2717	0.9960	0.0000	0.0309	0.7993
CCL4	turquoise	0.5035	0.0000	-0.5035	0.0000	-0.3840	0.0010	-0.3270	0.0057	-0.0137	0.9103	0.2812	0.0184	0.5145	0.0000
CLEC5A	turquoise	-0.4099	0.0004	0.4099	0.0004	0.0351	0.7730	0.3421	0.0038	-0.3564	0.0025	-0.2647	0.0268	0.0827	0.4959
CRYGB	turquoise	0.5828	0.0000	-0.5828	0.0000	-0.1268	0.2957	-0.0881	0.4685	0.1323	0.2750	0.9985	0.0000	0.0116	0.9243
CTXN3	turquoise	0.5836	0.0000	-0.5836	0.0000	-0.1447	0.2320	-0.1108	0.3613	0.1244	0.3048	0.9979	0.0000	0.0475	0.6962
DEFB125	turquoise	0.5843	0.0000	-0.5843	0.0000	-0.1626	0.1787	-0.1209	0.3189	0.1303	0.2824	0.9954	0.0000	0.0293	0.8098
DNAH14	turquoise	0.5945	0.0000	-0.5945	0.0000	-0.1308	0.2806	-0.0980	0.4195	0.1382	0.2540	0.9982	0.0000	0.0030	0.9805
EYA1	turquoise	0.5841	0.0000	-0.5841	0.0000	-0.1511	0.2118	-0.1092	0.3682	0.1210	0.3184	0.9973	0.0000	0.0484	0.6907
FAM24A	turquoise	0.5811	0.0000	-0.5811	0.0000	-0.1393	0.2499	-0.1034	0.3942	0.1297	0.2847	0.9975	0.0000	0.0369	0.7620
FAM47B	turquoise	0.5752	0.0000	-0.5752	0.0000	-0.1200	0.3223	-0.0931	0.4433	0.1288	0.2880	0.9943	0.0000	0.0211	0.8622
FGF20	turquoise	0.5875	0.0000	-0.5875	0.0000	-0.1508	0.2126	-0.1048	0.3881	0.1295	0.2853	0.9969	0.0000	0.0386	0.7510
FLJ37201	turquoise	0.5652	0.0000	-0.5652	0.0000	-0.1237	0.3076	-0.0944	0.4371	0.1243	0.3052	0.9970	0.0000	0.0121	0.9209
GDPD2	turquoise	0.5732	0.0000	-0.5732	0.0000	-0.1128	0.3523	-0.0888	0.4650	0.1324	0.2746	0.9974	0.0000	0.0114	0.9254
GRHL2	turquoise	0.5813	0.0000	-0.5813	0.0000	-0.1273	0.2936	-0.1023	0.3993	0.1384	0.2531	0.9980	0.0000	0.0191	0.8752
HCRTR2	turquoise	0.5940	0.0000	-0.5940	0.0000	-0.1307	0.2810	-0.1077	0.3747	0.1361	0.2613	0.9974	0.0000	0.0113	0.9258
HLA-DRB4	turquoise	-0.4278	0.0002	0.4278	0.0002	-0.1076	0.3752	-0.0415	0.7329	0.1361	0.2612	-0.2628	0.0280	-0.2882	0.0156
HOXA11	turquoise	0.5956	0.0000	-0.5956	0.0000	-0.1431	0.2372	-0.0946	0.4358	0.1237	0.3076	0.9987	0.0000	0.0350	0.7734
HS3ST3A1	turquoise	0.5806	0.0000	-0.5806	0.0000	-0.1310	0.2796	-0.1005	0.4076	0.1386	0.2524	0.9982	0.0000	0.0139	0.9093
IMPG1	turquoise	0.5910	0.0000	-0.5910	0.0000	-0.1566	0.1954	-0.1136	0.3492	0.1425	0.2391	0.9962	0.0000	0.0184	0.8799

KCNB2	turquoise	0.5944	0.0000	-0.5944	0.0000	-0.1472	0.2239	-0.0964	0.4274	0.1313	0.2788	0.9987	0.0000	0.0267	0.8265
KLK7	turquoise	0.5876	0.0000	-0.5876	0.0000	-0.1393	0.2503	-0.1067	0.3793	0.1275	0.2927	0.9992	0.0000	0.0234	0.8477
LCE1B	turquoise	0.5732	0.0000	-0.5732	0.0000	-0.1345	0.2668	-0.1051	0.3864	0.1190	0.3265	0.9970	0.0000	0.0273	0.8226
LGALS2	turquoise	-0.5079	0.0000	0.5079	0.0000	0.2553	0.0329	0.0020	0.9869	0.0065	0.9577	-0.3264	0.0058	-0.1185	0.3284
LOC100129884	turquoise	0.6050	0.0000	-0.6050	0.0000	-0.1442	0.2337	-0.1103	0.3635	0.1422	0.2401	0.9979	0.0000	0.0093	0.9388
LOC100131860	turquoise	0.5878	0.0000	-0.5878	0.0000	-0.1396	0.2491	-0.0965	0.4269	0.1485	0.2198	0.9977	0.0000	-0.0004	0.9971
LOC286071	turquoise	0.6035	0.0000	-0.6035	0.0000	-0.1330	0.2725	-0.0985	0.4171	0.1376	0.2561	0.9977	0.0000	0.0140	0.9085
LRRC3B	turquoise	0.5725	0.0000	-0.5725	0.0000	-0.1158	0.3399	-0.1136	0.3491	0.1222	0.3135	0.9966	0.0000	0.0281	0.8173
LRRC4C	turquoise	0.5955	0.0000	-0.5955	0.0000	-0.1397	0.2488	-0.1081	0.3729	0.1402	0.2470	0.9975	0.0000	0.0209	0.8637
MAGEA1	turquoise	0.5895	0.0000	-0.5895	0.0000	-0.1395	0.2494	-0.1171	0.3344	0.1301	0.2829	0.9974	0.0000	0.0288	0.8131
NPAS4	turquoise	0.5841	0.0000	-0.5841	0.0000	-0.1208	0.3193	-0.1069	0.3783	0.1209	0.3187	0.9984	0.0000	0.0269	0.8252
NPY2R	turquoise	0.5846	0.0000	-0.5846	0.0000	-0.1356	0.2629	-0.1024	0.3991	0.1240	0.3066	0.9989	0.0000	0.0362	0.7664
PHOX2B	turquoise	0.5938	0.0000	-0.5938	0.0000	-0.1285	0.2892	-0.1041	0.3912	0.1236	0.3081	0.9981	0.0000	0.0343	0.7783
PODN	turquoise	0.5914	0.0000	-0.5914	0.0000	-0.1457	0.2289	-0.1053	0.3856	0.1229	0.3108	0.9988	0.0000	0.0350	0.7738
POLN	turquoise	0.5895	0.0000	-0.5895	0.0000	-0.1402	0.2470	-0.1117	0.3572	0.1369	0.2583	0.9989	0.0000	0.0213	0.8612
RAMP2	turquoise	0.5904	0.0000	-0.5904	0.0000	-0.1362	0.2609	-0.0896	0.4605	0.1136	0.3489	0.9978	0.0000	0.0348	0.7747
RASSF9	turquoise	0.5818	0.0000	-0.5818	0.0000	-0.1277	0.2922	-0.0885	0.4662	0.1247	0.3039	0.9986	0.0000	0.0093	0.9388
S100A7A	turquoise	0.6056	0.0000	-0.6056	0.0000	-0.1578	0.1919	-0.1049	0.3876	0.1366	0.2594	0.9985	0.0000	0.0245	0.8406
SCARNA17	turquoise	0.4497	0.0001	-0.4497	0.0001	-0.2688	0.0244	-0.4115	0.0004	0.3823	0.0011	0.2604	0.0294	-0.0302	0.8042
SFTA3	turquoise	0.5919	0.0000	-0.5919	0.0000	-0.1472	0.2240	-0.0968	0.4254	0.1338	0.2696	0.9974	0.0000	0.0022	0.9854
SLC22A25	turquoise	0.5798	0.0000	-0.5798	0.0000	-0.1293	0.2859	-0.0967	0.4259	0.1259	0.2989	0.9990	0.0000	0.0161	0.8948
SLC25A18	turquoise	0.6090	0.0000	-0.6090	0.0000	-0.1564	0.1959	-0.1123	0.3547	0.1227	0.3116	0.9975	0.0000	0.0348	0.7752
SLC25A31	turquoise	0.6026	0.0000	-0.6026	0.0000	-0.1323	0.2751	-0.0986	0.4169	0.1251	0.3021	0.9961	0.0000	-0.0042	0.9722
SLC6A3	turquoise	0.5889	0.0000	-0.5889	0.0000	-0.1319	0.2764	-0.0946	0.4361	0.1228	0.3113	0.9988	0.0000	0.0267	0.8263
SLCO1B1	turquoise	0.6113	0.0000	-0.6113	0.0000	-0.1378	0.2553	-0.1106	0.3621	0.1256	0.3001	0.9969	0.0000	0.0322	0.7912
SOX14	turquoise	0.5838	0.0000	-0.5838	0.0000	-0.1439	0.2347	-0.1156	0.3408	0.1298	0.2842	0.9965	0.0000	0.0350	0.7738

SOX6	turquoise	0.5959	0.0000	-0.5959	0.0000	-0.1377	0.2558	-0.1025	0.3983	0.1326	0.2737	0.9994	0.0000	0.0221	0.8558
SPEM1	turquoise	0.5845	0.0000	-0.5845	0.0000	-0.1121	0.3554	-0.0918	0.4496	0.1347	0.2662	0.9979	0.0000	0.0150	0.9018
TAS2R1	turquoise	0.5888	0.0000	-0.5888	0.0000	-0.1358	0.2624	-0.1168	0.3357	0.1355	0.2632	0.9978	0.0000	0.0225	0.8531
TEKT1	turquoise	0.6069	0.0000	-0.6069	0.0000	-0.1633	0.1769	-0.1047	0.3882	0.1309	0.2799	0.9977	0.0000	0.0414	0.7335
THAP9	turquoise	0.5881	0.0000	-0.5881	0.0000	-0.1710	0.1570	-0.0986	0.4167	0.1221	0.3138	0.9930	0.0000	0.0322	0.7911
TRPC5	turquoise	0.5984	0.0000	-0.5984	0.0000	-0.1520	0.2091	-0.1009	0.4061	0.1227	0.3116	0.9979	0.0000	0.0139	0.9090
UGT2A3	turquoise	0.5843	0.0000	-0.5843	0.0000	-0.1332	0.2718	-0.1064	0.3806	0.1274	0.2932	0.9965	0.0000	0.0040	0.9736
UNC13C	turquoise	0.5850	0.0000	-0.5850	0.0000	-0.1360	0.2616	-0.1065	0.3803	0.1239	0.3068	0.9990	0.0000	0.0291	0.8111
VWC2	turquoise	0.5931	0.0000	-0.5931	0.0000	-0.0962	0.4284	-0.0864	0.4769	0.1407	0.2454	0.9910	0.0000	-0.0335	0.7832
ZIC1	turquoise	0.5935	0.0000	-0.5935	0.0000	-0.1263	0.2975	-0.1088	0.3702	0.1226	0.3121	0.9983	0.0000	0.0149	0.9026
ANXA3	yellow	-0.0032	0.9787	0.0032	0.9787	0.2933	0.0137	0.6922	0.0000	-0.3338	0.0047	-0.0255	0.8338	-0.1135	0.3497
AZU1	yellow	0.0680	0.5757	-0.0680	0.5757	0.2423	0.0433	0.7547	0.0000	-0.0812	0.5040	0.0243	0.8416	-0.1791	0.1380
CAMP	yellow	-0.1772	0.1421	0.1772	0.1421	0.3864	0.0010	0.9285	0.0000	-0.3435	0.0036	-0.1162	0.3380	-0.1609	0.1833
CEACAM6	yellow	-0.1022	0.3997	0.1022	0.3997	0.2217	0.0651	0.9268	0.0000	-0.2788	0.0194	-0.0818	0.5007	-0.0439	0.7182
CEACAM8	yellow	-0.0570	0.6391	0.0570	0.6391	0.3025	0.0109	0.9535	0.0000	-0.2107	0.0799	-0.0746	0.5393	-0.1271	0.2943
CRISP3	yellow	-0.0646	0.5951	0.0646	0.5951	0.1972	0.1018	0.6558	0.0000	-0.0115	0.9248	-0.1745	0.1486	-0.2094	0.0819
DEFA4	yellow	-0.0465	0.7023	0.0465	0.7023	0.1755	0.1462	0.8590	0.0000	-0.1190	0.3265	-0.0372	0.7596	-0.0931	0.4435
HP	yellow	-0.0203	0.8677	0.0203	0.8677	0.2248	0.0613	0.4549	0.0001	-0.3471	0.0032	-0.0294	0.8088	-0.1940	0.1076
LCN2	yellow	-0.1095	0.3670	0.1095	0.3670	0.5105	0.0000	0.9027	0.0000	-0.2814	0.0183	-0.0760	0.5315	-0.2119	0.0782
LTF	yellow	-0.0373	0.7589	0.0373	0.7589	0.4262	0.0002	0.9359	0.0000	-0.2497	0.0371	-0.0347	0.7757	-0.1775	0.1416
MMP8	yellow	-0.2409	0.0446	0.2409	0.0446	0.2716	0.0230	0.7379	0.0000	-0.1161	0.3386	-0.1713	0.1563	-0.1263	0.2975
MS4A3	yellow	-0.1250	0.3026	0.1250	0.3026	0.1262	0.2977	0.6041	0.0000	-0.0022	0.9853	-0.0868	0.4748	-0.0158	0.8968
OLFM4	yellow	-0.2049	0.0888	0.2049	0.0888	0.2939	0.0135	0.8639	0.0000	-0.2127	0.0772	-0.2240	0.0623	-0.2332	0.0520
S100P	yellow	0.0572	0.6382	-0.0572	0.6382	0.2305	0.0549	0.5431	0.0000	-0.2689	0.0244	0.0300	0.8054	0.2012	0.0949
TCN1	yellow	-0.2438	0.0419	0.2438	0.0419	0.2594	0.0302	0.7367	0.0000	-0.2036	0.0909	-0.1562	0.1966	-0.0268	0.8254

Table S3 Demographic and clinical data of the validation population and controls.

Here we have provided an exhaustive demographic and clinical data of the validation population and controls.

CCL4

Patients	Age	Gender	Clinical Phenotype
BD1	21	Female	Mucocutaneous manifestations; Gastrointestinal involvement
BD2	29	Male	Mucocutaneous manifestations; Ocular involvement; Joint involvement; Vascular involvement
BD3	23	Male	Vascular involvement; Mucocutaneous manifestations; Gastrointestinal involvement
BD4	33	Male	Vascular involvement; Mucocutaneous manifestations
BD5	45	Male	Vascular involvement
BD6	31	Male	Mucocutaneous manifestations; Ocular involvement
BD7	32	Female	Vascular involvement; Mucocutaneous manifestations
BD8	57	Female	Mucocutaneous manifestations; Gastrointestinal involvement
BD9	44	Male	Vascular involvement; Mucocutaneous manifestations
BD10	46	Male	Vascular involvement; Mucocutaneous manifestations
BD11	21	Male	Vascular involvement; Mucocutaneous manifestations
BD12	44	Male	Vascular involvement; Mucocutaneous manifestations
BD13	30	Male	Vascular involvement; Mucocutaneous manifestations
BD14	58	Male	Mucocutaneous manifestations; Gastrointestinal involvement
BD15	56	Female	Mucocutaneous manifestations; Gastrointestinal involvement
BD16	24	Female	Mucocutaneous manifestations; Neurological involvement

NPY2R

Patients	Age	Gender	Involvement
BD1	29	Male	Mucocutaneous manifestations; Ocular involvement; Joint involvement; Vascular involvement
BD2	23	Male	Vascular involvement; Mucocutaneous manifestations; Gastrointestinal involvement
BD3	33	Male	Vascular involvement; Mucocutaneous manifestations
BD4	45	Male	Vascular involvement
BD5	31	Male	Mucocutaneous manifestations; Ocular involvement
BD6	32	Female	Vascular involvement; Mucocutaneous manifestations
BD7	57	Female	Mucocutaneous manifestations; Gastrointestinal involvement
BD8	44	Male	Vascular involvement; Mucocutaneous manifestations
BD9	46	Male	Vascular involvement; Mucocutaneous manifestations
BD10	21	Male	Vascular involvement; Mucocutaneous manifestations
BD11	44	Male	Vascular involvement; Mucocutaneous manifestations
BD12	30	Male	Vascular involvement; Mucocutaneous manifestations
BD13	58	Male	Mucocutaneous manifestations; Gastrointestinal involvement
BD14	24	Female	Mucocutaneous manifestations; Neurological involvement
BD15	37	Male	Vascular involvement; Mucocutaneous manifestations
BD16	29	Male	Vascular involvement; Mucocutaneous manifestations

CCL4

Patients	Age	Gender	Medical Records	Medication within 3 months
BD1	21	Female	Recurrent oral ulcers, vulvar ulcers, appendicitis, ileocecal valve ulcers	Colchicine Sulfasalazine
BD2	29	Male	2018: Repeated oral ulcers, multiple penile ulcers ;<br>2020: Epididymitis, anterior uveitis; slight joint pain;<br>2021.1: worsened knee joint pain, twice epididymitis, superficial phlebitis on the medial right ankle with thrombosis	Adalimumab Prednisone Methotrexate Aspirin
BD3	23	Male	2020.8 Femoral artery aneurysm with thrombosis; intestinal perforation, oral ulcer, vulvar ulcer, new scrotal ulcer;<br>2020.11.10 tuberculosis infection	Prednisone Mycophenolate mofetil Colchicine Leflunomide
BD4	33	Male	Previous venous thrombosis of the lower extremities and venous sinus thrombosis for 3 years;<br>2020.10 recurrent oral ulcers, folliculitis, left sigmoid sinus and left popliteal vein stenosis, left epididymal head cyst, no headache, vision loss, posterior left lower extremity and intracranial venous sinus thrombosis	Colchicine Leflunomide Azathioprine
BD5	45	Male	6+ years after aneurysm surgery	None
BD6	31	Male	Repeated oral and vulvar painful ulcers, folliculitis, uveitis in early 2020; history of tuberculosis in 2019	Intermittent administration of Thalidomide
BD7	32	Female	Recurrent oral ulcers for 2 years, vulvar ulcers;<br>2020.10.25 Right common iliac and external iliac vein thrombosis;<br>2021.6.24 Intracranial venous sinus thrombosis, with menstrual single oral ulcers	2021.6.16 Baricitinib (JAK inhibitor)<br>2021.9.1 Baricitinib<br>Colchicine<br>Azathioprine<br>Prednisone
BD8	57	Female	Recurrent oral ulcers, 2-3 times a year, once had a vulvar ulcer, no lower limb erythema. Chronic enteritis of the mucosa in 2017, a large number of inflammatory cell infiltration, a large number of inflammatory exudates and necrotic tissue; ileal colon ulcer, lymphoid tissue hyperplasia.	Cyclophosphamide Tacrolimus Prednisone Ursodeoxycholic acid
BD9	44	Male	2019.4 thrombosis in the deep vein (popliteal vein) of the right lower extremity; Repeated oral ulcers, no vulvar ulcers, no red eyes and eye pain. 2019.4: Stasis dermatitis. No pulmonary embolism, the main inferior vena cava below the level of the right renal vein is unclear, and there are multiple tortuous vascular shadows around, low-density filling defects can be seen in the superficial femoral veins on both sides, and the superficial veins of the lower limbs are tortuous and dilated.	Aescuvenforte Prednisone Mycophenolate mofetil

BD10	46	Male	Systemic vasculitis plus Behcet's disease, no oral and vulvar ulcers.<br>2012 Skin pustules, acne-like rash of the head and neck;<br>2014.3 Diffuse metabolism of the aortic wall was increased, considered as vasculitis;<br>2015 Thickening and stenosis of the aorta;<br>2019 Thickening of the aortic wall and severe stenosis of the arterial lumen	Methotrexate<br>Azathioprine<br>Aspirin
BD11	21	Male	Repeated oral ulcers for more than 7 years, folliculitis, painful erythema of the lower limbs, no vulvar ulcer; thrombosis of the superficial vein of the left lower limb.	Thalidomide<br>Colchicine
BD12	44	Male	Takayasu arteritis plus Behcet's disease. Repeated oral ulcers for 6 years, no vulvar ulcers, no uveitis, no erythema nodosa; aortic insufficiency, thickening and prolapse; thickening of the ascending aorta, intimal stratification, suspected aortic arteritis,<br>2021.2 paravalvular Leak replacement surgery, obvious alterations in inflammatory adhesions around the aorta, aortic valve thickening and stratification, consistent with inflammatory changes	Prednisone<br>Hydroxychloroquine sulfate<br>Mycophenolate mofetil<br>Ursodeoxycholic acid<br>Cyclophosphamide
BD13	30	Male	Repeated oral ulcers for 14 years without vulvar ulcers, no erythema nodules, iritis, or diarrhea. There is folliculitis on the back of the neck.<br>2021.6 Left lower limb venous thrombosis for 6 months, varicose veins with thrombophlebitis in left abdominal superficial for 3 weeks;<br>2021.8 tuberculosis infection	2021.6 Rivaroxaban;<br>2021.7 Enoxaparin sodium injection<br>Prednisone<br>Mycophenolate mofetil<br>dexamethasone;<br>2021.9 Adalimumab<br>Prednisone acetate<br>Cyclophosphamide<br>Colchicine<br>Rivaroxaban
BD14	58	Male	Intestinal involvement in Behcet's disease: repeated oral ulcers for 2 years, denial of vulvar ulcers, skin nodules, blurred vision; intermittent abdominal pain for more than 1 year, colonoscopy showed: acute and chronic inflammation of the ascending colon mucosa, focal erosion, multiple small ulcers in the terminal and entire colon, pathology: severe chronic inflammation of the mucosa, accompanied by active inflammation, visible of ulcers.<br>2020.1 Right eyelid inflammation;<br>2021.5 terminal ileum ulcer and erosion, no oral vulvar ulcer	2021.5 Thalidomide<br>Total glucosides of paeony<br>Sulfasalazine<br>Prednisone<br>Tacrolimus;<br>2021.9 Cyclosporine<br>Thalidomide<br>Total glucosides of paeony<br>Sulfasalazine<br>Prednisone
BD15	56	Female	Behcet's disease plus esophageal ulcer. Repeated oral ulcers, vulvar ulcers, perianal ulcers.<br>2020.9 Upper abdominal discomfort, gastroscopy: three ulcers in the middle and lower esophagus, pathology: mucosal inflammation with exudation and necrosis; overlying squamous epithelial hyperplasia, interstitial focal lymphocytic hyperplasia, and granulation	Azathioprine<br>Prednisone<br>Total glucosides of paeony

			tissue.	Levothyroxin Sodium
BD16	24	Female	2021.2 Fever+ lymphadenectasis+ painless oral ulcer + vulvar ulcer, no rash, arthralgia and other symptoms;<br>2021 Central Nervous System midline and temporal lobe lesions for more than 3 months, encephalitis + brain brainstem encephalitis	2021.6<br>Mycophenolate mofetil<br>Total glucosides of paeony<br>Colchicine

NPY2R

Patients	Age	Gender	Medical Records	Medication within 3 months
BD1	29	Male	2018: Repeated oral ulcers, multiple penile ulcers ;<br>2020: Epididymitis, anterior uveitis; slight joint pain;<br>2021.1: worsened knee joint pain, twice epididymitis, superficial phlebitis on the medial right ankle with thrombosis	Adalimumab<br>Prednisone<br>Methotrexate<br>Aspirin
BD2	23	Male	2020.8 Femoral artery aneurysm with thrombosis; intestinal perforation, oral ulcer, vulvar ulcer, new scrotal ulcer;<br>2020.11.10 tuberculosis infection	Prednisone<br>Mycophenolate mofetil<br>Colchicine<br>Leflunomide
BD3	33	Male	Previous venous thrombosis of the lower extremities and venous sinus thrombosis for 3 years;<br>2020.10 recurrent oral ulcers, folliculitis, left sigmoid sinus and left popliteal vein stenosis, left epididymal head cyst, no headache, vision loss, posterior left lower extremity and intracranial venous sinus thrombosis	Colchicine<br>Leflunomide<br>Azathioprine
BD4	45	Male	6+ years after aneurysm surgery	None
BD5	31	Male	Repeated oral and vulvar painful ulcers, folliculitis, uveitis in early 2020; history of tuberculosis in 2019	Intermittent administration of Thalidomide
BD6	32	Female	Recurrent oral ulcers for 2 years, vulvar ulcers;<br>2020.10.25 Right common iliac and external iliac vein thrombosis;<br>2021.6.24 Intracranial venous sinus thrombosis, with menstrual single oral ulcers	2021.6.16 Baricitinib (JAK inhibitor)<br>2021.9.1 Baricitinib<br>Colchicine<br>Azathioprine<br>Prednisone
BD7	57	Female	Recurrent oral ulcers, 2-3 times a year, once had a vulvar ulcer, no lower limb erythema. Chronic enteritis of the mucosa in 2017, a large number of inflammatory cell infiltration, a large number of inflammatory exudates and necrotic tissue; ileal colon ulcer, lymphoid tissue hyperplasia.	Cyclophosphamide<br>Tacrolimus<br>Prednisone<br>Ursodeoxycholic acid

BD8	44	Male	2019.4 thrombosis in the deep vein (popliteal vein) of the right lower extremity; Repeated oral ulcers, no vulvar ulcers, no red eyes and eye pain. 2019.4: Stasis dermatitis. No pulmonary embolism, the main inferior vena cava below the level of the right renal vein is unclear, and there are multiple tortuous vascular shadows around, low-density filling defects can be seen in the superficial femoral veins on both sides, and the superficial veins of the lower limbs are tortuous and dilated.	Aescuvenforte<br>Prednisone<br>Mycophenolate mofetil
BD9	46	Male	Systemic vasculitis plus Behcet's disease, no oral and vulvar ulcers.<br>2012 Skin pustules, acne-like rash of the head and neck;<br>2014.3 Diffuse metabolism of the aortic wall was increased, considered as vasculitis;<br>2015 Thickening and stenosis of the aorta;<br>2019 Thickening of the aortic wall and severe stenosis of the arterial lumen	Methotrexate<br>Azathioprine<br>Aspirin
BD10	21	Male	Repeated oral ulcers for more than 7 years, folliculitis, painful erythema of the lower limbs, no vulvar ulcer; thrombosis of the superficial vein of the left lower limb.	Thalidomide<br>Colchicine
BD11	44	Male	Takayasu arteritis plus Behcet's disease. Repeated oral ulcers for 6 years, no vulvar ulcers, no uveitis, no erythema nodosa; aortic insufficiency, thickening and prolapse; thickening of the ascending aorta, intimal stratification, suspected aortic arteritis,<br>2021.2 paravalvular Leak replacement surgery, obvious alterations in inflammatory adhesions around the aorta, aortic valve thickening and stratification, consistent with inflammatory changes	Prednisone<br>Hydroxychloroquine sulfate<br>Mycophenolate mofetil<br>Ursodeoxycholic acid<br>Cyclophosphamide
BD12	30	Male	Repeated oral ulcers for 14 years without vulvar ulcers, no erythema nodules, iritis, or diarrhea. There is folliculitis on the back of the neck.<br>2021.6 Left lower limb venous thrombosis for 6 months, varicose veins with thrombophlebitis in left abdominal superficial for 3 weeks;<br>2021.8 tuberculosis infection	2021.6<br>Rivaroxaban
BD13	58	Male	Intestinal involvement in Behcet's disease: repeated oral ulcers for 2 years, denial of vulvar ulcers, skin nodules, blurred vision; intermittent abdominal pain for more than 1 year, colonoscopy showed: acute and chronic inflammation of the ascending colon mucosa, focal erosion, multiple small ulcers in the terminal and entire colon, pathology: severe chronic inflammation of the mucosa, accompanied by active inflammation, visible of ulcers.<br>2020.1 Right eyelid inflammation;<br>2021.5 terminal ileum ulcer and erosion, no oral vulvar ulcer	2021.7 Enoxaparin sodium injection<br>Prednisone<br>Mycophenolate mofetil<br>dexamethasone;
BD14	24	Female	2021.2 Fever+ lymphadenectasis +painless oral ulcer +vulvar ulcer, no rash, arthralgia and other symptoms;<br>2021 Central Nervous System midline and temporal lobe lesions for more than 3 months, encephalitis + brainstem encephalitis	2021.9 Adalimumab<br>Prednisone acetate<br>Cyclophosphamide<br>Colchicine<br>Rivaroxaban"
BD15	37	Male	History of tuberculosis;<br>2014 right internal carotid artery dissecting aneurysm;<br>2017 right internal carotid artery occlusion; 2020.8 Deep Vein Thrombosis of right calf, pulmonary embolism; intermittent oral ulcer, 10+ times/year, denying vulvar ulcer and pathergy reaction, occasional skin furuncle. No joint swelling and pain, no uveitis;	2021.5<br>Thalidomide<br>Total glucosides of paeony<br>Sulfasalazine

			2021.6 right common femoral artery pseudoaneurysm with thrombosis	Prednisone Tacrolimus;
BD16	29	Male	Myocardial involvement: history of appendicitis; repeated oral ulcers and once vulvar ulcer; multiple pulmonary artery occlusion in the right lung, which was considered chronic pulmonary embolism, vasculitis may get involved; right ventricular filling defect, consider the possibility of large thrombus; multiple lungs patches, consolidation and mass shadows, with multiple cavities, granulomatous inflammation; intraventricular thrombosis. Superior sagittal sinus, sinus confluence, right transverse sinus, sigmoid sinus stenosis/occlusion; left subclavian artery stenosis; double optic papillary edema.	2021.9 Cyclosporine Thalidomide Total glucosides of paeony Sulfasalazine Prednisone "

Healthy controls are **age- and sex-matched** individuals from physical examination center who are selected according to the criteria (i) all of the serum biochemical indicators within the normal reference interval; (ii) no medical history of chronic diseases or serious disease conditions such as cardiovascular, liver, kidney, blood and lymph, endocrine, immune, neuromuscular, gastrointestinal system, etc. within three years.

Healthy Control	Age	Gender
HC1	37	Male
HC2	37	Male
HC3	28	Male
HC4	37	Male
HC5	34	Male
HC6	28	Female
HC7	51	Female
HC8	36	Female
HC9	50	Male
HC10	36	Male
HC11	27	Male
HC12	58	Male
HC13	39	Male
HC14	42	Male
HC15	57	Female
HC16	45	Male

References

1. Ricciardi L, Giurato G, Memoli D, et al. Posttranscriptional Gene Regulatory Networks in Chronic Airway Inflammatory Diseases: In silico Mapping of RNA-Binding Protein Expression in Airway Epithelium. *Front Immunol* (2020) 11:579889. Epub 2020/11/13. doi: 10.3389/fimmu.2020.579889.
2. Huang J, Liu L, Qin L, et al. Weighted Gene Coexpression Network Analysis Uncovers Critical Genes and Pathways for Multiple Brain Regions in Parkinson's Disease. *Biomed Res Int* (2021) 2021:6616434. Epub 2021/04/02. doi: 10.1155/2021/6616434.
3. Zhang H, Zhang J, Xu Q, et al. Identification of candidate tolerance genes to low-temperature during maize germination by GWAS and RNA-seqapproaches. *BMC Plant Biol* (2020) 20(1):333. Epub 2020/07/16. doi: 10.1186/s12870-020-02543-9.
4. Liang W, Sun F, Zhao Y, et al. Identification of Susceptibility Modules and Genes for Cardiovascular Disease in Diabetic Patients Using WGCNA Analysis. *J Diabetes Res* (2020) 2020:4178639. Epub 2020/05/27. doi: 10.1155/2020/4178639.
5. Szklarczyk D, Gable AL, Lyon D, et al. STRING v11: protein-protein association networks with increased coverage, supporting functional discovery in genome-wide experimental datasets. *Nucleic Acids Res* (2019) 47(D1):D607-d13. Epub 2018/11/27. doi: 10.1093/nar/gky1131.
6. Bai Q, Liu H, Guo H, et al. Identification of Hub Genes Associated With Development and Microenvironment of Hepatocellular Carcinoma by Weighted Gene Co-expression Network Analysis and Differential Gene Expression Analysis. *Front Genet* (2020) 11:615308. Epub 2021/01/09. doi: 10.3389/fgene.2020.615308.
7. Thul PJ, Lindskog C. The human protein atlas: A spatial map of the human proteome. *Protein Sci* (2018) 27(1):233-44. Epub 2017/09/25. doi: 10.1002/pro.3307.
8. Zhang Y, Yang X, Zhu XL, et al. Bioinformatics analysis of potential core genes for glioblastoma. *Biosci Rep* (2020) 40(7). Epub 2020/07/16. doi: 10.1042/bsr20201625.
9. Liu J, Zhou S, Li S, et al. Eleven genes associated with progression and prognosis of endometrial cancer (EC) identified by comprehensive bioinformatics analysis. *Cancer Cell Int* (2019) 19:136. Epub 2019/05/30. doi: 10.1186/s12935-019-0859-1.
10. Guo A, Wang W, Shi H, et al. Identification of Hub Genes and Pathways in a Rat Model of Renal Ischemia-Reperfusion Injury Using Bioinformatics Analysis of the Gene Expression Omnibus (GEO) Dataset and Integration of Gene Expression Profiles. *Med Sci Monit* (2019) 25:8403-11. Epub 2019/11/09. doi: 10.12659/msm.920364.
